# metaFARVAT: An Efficient Tool for Meta-Analysis of Family-Based, Case-Control, and Population-Based Rare Variant Association Studies

**DOI:** 10.3389/fgene.2019.00572

**Published:** 2019-06-19

**Authors:** Longfei Wang, Sungyoung Lee, Dandi Qiao, Michael H. Cho, Edwin K. Silverman, Christoph Lange, Sungho Won

**Affiliations:** ^1^Interdisciplinary Program in Bioinformatics, Seoul National University, Seoul, South Korea; ^2^Center for Precision Medicine, Seoul National University Hospital, Seoul, South Korea; ^3^Channing Division of Network Medicine, Department of Medicine, Brigham and Women's Hospital and Harvard Medical School, Boston, MA, United States; ^4^Division of Pulmonary and Critical Care Medicine, Brigham and Women's Hospital, Boston, MA, United States; ^5^Department of Biostatistics, Harvard T.H. Chan School of Public Health, Boston, MA, United States; ^6^Department of Public Health Sciences, Seoul National University, Seoul, South Korea; ^7^Institute of Health and Environment, Seoul National University, Seoul, South Korea

**Keywords:** meta-analysis, family-based design, rare variant association analysis, metaFARVAT, chronic obstructive pulmonary disease

## Abstract

Family-based designs have been shown to be powerful in detecting the significant rare variants associated with human diseases. However, very few significant results have been found owing to relatively small sample sizes and the fact that statistical analyses often suffer from high false-negative error rates. These limitations can be avoided by combining results from multiple studies via meta-analysis. However, statistical methods for meta-analysis with rare variants are limited for family-based samples. In this report, we propose a tool for the meta-analysis of family-based rare variant associations, metaFARVAT. metaFARVAT is based on a quasi-likelihood score for each variant. These scores are combined to generate burden test, variable-threshold test, sequence kernel association test (SKAT), and optimal SKAT statistics. The proposed method tests homogeneous and heterogeneous effects of variants among different studies and can be applied to both quantitative and dichotomous phenotypes. Simulation results demonstrated the robustness and efficiency of the proposed method in different scenarios. By applying metaFARVAT to data from a family-based study and a case-control study, we identified a few promising candidate genes, including *DLEC1*, which is associated with chronic obstructive pulmonary disease.

## Introduction

In recent decades, genome-wide association studies (GWAS) have identified tens of thousands of common variants associated with various complex diseases. However, in spite of their success in discovering disease susceptibility loci (DSL), the DSL identified by GWAS only partially explain disease heritability, and rare variants have been implicated as one contributor to this missing heritability (Manolio et al., 2009). Recent improvements in sequencing technology have enabled rare variant association analyses, and various methods have been proposed for rare variant association studies, such as the combined multivariate and collapsing (CMC) method, burden test, variable-threshold test (VT) (Price et al., 2010), sequence kernel association test (SKAT) (Wu et al., 2011), and optimal SKAT (SKAT-O) (Lee et al., 2012b).

Multiple rare variants can affect disease status, and thus, association analyses with rare variants suffer from genetic heterogeneity among affected individuals. In families, Mendelian transmission results in family members sharing the same alleles, and therefore, affected relatives have a greater chance of being affected by the same disease-causing single-nucleotide polymorphisms (SNPs) than unrelated subjects. For instance, the probability of sibling pairs sharing rare alleles can be calculated (Ionita-Laza et al., 2011). Therefore, family-based analyses have been generally recognized as an important strategy for rare variant association studies. We proposed FARVAT (Choi et al., 2014) statistics based on quasi-likelihood. This includes burden, SKAT, and SKAT-O statistics for both dichotomous and quantitative phenotypes, and we have shown that they are robust against population substructure and outperform other existing rare variant association tests for family samples (Wang et al., 2016).

Aggregation of association signals across multiple genetic variants was expected to provide sufficient statistical power for rare variant analyses and to identify various DSL. However, very few genome-wide significant results have been found because of relatively small sample sizes. When the sample size is small, statistical analyses suffer from high false-negative error rates, and this limitation can be avoided by combining results from multiple studies via mega- or meta-analysis. Mega-analysis assumes that subjects' genotypes and phenotypes from different studies are available, and these are pooled for genetic association analyses. Meta-analysis directly utilizes test statistics from separate studies and combines them into a single test statistic. The choice between mega- and meta-analysis depends on the heterogeneity among studies and the availability of individual genotype and phenotype data from all studies. In particular, if there are systematic differences in phenotype diagnosis or sequencing technology, meta-analysis is often preferred. Otherwise, mega-analysis is recommended if genotypes and phenotypes are available. Recently, several meta-analysis methods for rare variant association tests have been proposed, such as MASS (Tang and Lin, 2013, 2014), RAREMETAL (Feng et al., 2014; Liu et al., 2014), seqMeta (Chen et al., 2014), and MetaSKAT (Lee et al., 2013). However, the available statistical methods for family-based samples or dichotomous phenotypes are limited, and it is moreover worthwhile to provide a method that can be applied to both quantitative and dichotomous phenotypes under homogeneous and heterogeneous disease models.

In this study, we proposed a new meta-analysis method for family-based, population-based, and case-control rare variant association tests, metaFARVAT. metaFARVAT generates a quasi-likelihood score for each variant and combines them to generate burden, VT, SKAT, and SKAT-O statistics. metaFARVAT can assume homogeneous or heterogeneous effects of variants among different studies and can be applied to both quantitative and dichotomous phenotypes. We evaluated the statistical validity of metaFARVAT using simulated data and compared its estimated power with those of RAREMETAL and seqMeta under various scenarios. Furthermore, metaFARVAT was applied to identify rare variants for chronic obstructive pulmonary disease (COPD) using whole-exome sequencing (WES) data from family-based samples from the Boston Early-Onset COPD Study (EOCOPD) and case-control samples from the COPDGene study.

## Methods

### Notations and Disease Model

We assume that there are *K* studies available and that each study is of either a population-based, case-control, or family-based design. It is assumed that *N_k_* subjects are available in study *k*. We assume that there are *M* rare variants in a gene, and the minor allele count of variant *m* for subject *i* in study *k* is coded by *x_imk_*. Traits can be either quantitative or dichotomous, and *y_ik_* indicates a phenotype of subject *i* in study *k*. Their vectors are denoted by:

$$\begin{array}{l} {\text{X}}_{k}^{j}=\left[\begin{array}{c} x_{1jk}\\ \vdots \\ x_{N_{k}jk} \end{array}\right],\;{\text{X}}_{k}=\left({\text{X}}_{k}^{1},\;\cdots \;,\;{\text{X}}_{k}^{M}\right),\;{\text{Y}}_{k}=\left[\begin{array}{c} y_{1k}\\ \vdots \\ y_{N_{k}k} \end{array}\right]. \end{array}$$

In some cases, rare variants may be observed only in a subset of studies. If variant *m* is missing or monomorphic in study *k*, we assume that ${\text{X}}_{k}^{m}$ is **0**, and its variance and covariance with ${\text{X}}_{k}^{m^{\prime }}\left(m\neq m^{\prime }\right)\;$are 0. If variant *m* is missing for all studies, then it should be removed from the analysis.

Parental genotypes are transmitted to offspring under Mendelian transmission, and thus our test statistics consider the genetic correlation between family members. The genetic variance-covariance matrix among family members can be specified by a kinship coefficient matrix, **Φ**_*k*_. If we let $\pi_{i,i^{\prime },k}$ be the kinship coefficient between subject *i* and subject *i'* for study *k*, and *d_ik_* be the inbreeding coefficient for subject *i*, **Φ**_*k*_ is defined by:

$$\begin{array}{l} \left[\begin{array}{cc} \begin{array}{cc} \begin{array}{c} 1+d_{1,k}\\ 2\pi_{1,2,k} \end{array} & \begin{array}{c} 2\pi_{1,2,k}\\ 1+d_{2,k} \end{array} \end{array} & \begin{array}{cc} \begin{array}{c} 2\pi_{1,3,k}\\ 2\pi_{2,3,k} \end{array} & \begin{array}{c} \ldots \\ \ldots \end{array} \end{array}\\ \begin{array}{cc} \begin{array}{c} 2\pi_{1,3,k}\\ \vdots \end{array} & \begin{array}{c} \;2\pi_{2,3,k}\\ \vdots \end{array} \end{array} & \begin{array}{cc} \begin{array}{c} 1+d_{3,k}\\ \ddots \end{array} & \begin{array}{c} \ddots \\ \ddots \end{array} \end{array} \end{array}\right] \end{array}$$

Under the presence of population substructure, the genetic relationship matrix (GRM) can be estimated with large-scale genotyping data and should alternatively replace **Φ**_*k*_ (Thornton et al., 2012).

Last, meta-analysis of rare variant association analyses with multiple studies requires two different types of weights. First, when multiple studies are combined, each study has different features, such as sample size and disease diagnosis, and such differences can be handled with an *a priori* specified weight for each study. We assume that the statistics for study *k* are weighted by *v*_*k*_, and their *K* × *K* dimensional diagonal matrix is denoted by **W**_*B*_. Second, rare variants have different gene annotations, genomic coordinates, and functional characterization, and various annotation tools have been proposed to choose important features based on their biological properties. We denote the weight for rare variant *m* by *w_m_*, and we let **W**_*W*_ be their *M* × *M* dimensional diagonal matrix.

### Choices of Offset

We introduce the offset μ_*ik*_ for subject *i* at study *k* to improve the efficiency of the proposed score test (Lange et al., 2002). We set:

$$\begin{array}{l} \mu_{k}=\left[\begin{array}{c} \mu_{1k}\\ \vdots \\ \mu_{N_{k}k} \end{array}\right],\;\mu =\left(\mu_{1},\;\cdots \;,\;\mu_{K}\right),\;{\text{T}}_{k}={\text{Y}}_{k}-\mu_{k}. \end{array}$$

The most efficient choice of **μ** may depend on the sampling scheme, and either the best linear unbiased predictor (BLUP) with covariates or the prevalence were shown to be the most efficient (Won and Lange, 2013). If families are randomly selected, BLUP was shown to be the most efficient (Won and Lange, 2013); otherwise, the prevalence is recommended for dichotomous phenotypes (Thornton and McPeek, 2007; Won and Lange, 2013). In this report, we focus on randomly selected families, and we incorporate BLUP from the linear mixed model for **μ**. Under the null hypothesis, the linear mixed model (George and Elston, 1987) for a quantitative phenotype is given by:

$$\begin{array}{l} \text{Y}=\text{Z}\alpha +\text{B}+\text{E},\;\text{B}\sim MVN\left(0,\sigma_{b}^{2}\Phi \right)\;and\;\text{E}\sim MVN\left(0,\sigma_{e}^{2}{\text{I}}_{N}\right), \end{array}$$

where **B** and **E** indicate the polygenetic random effect and random error, respectively. Then, incorporation of BLUP as an offset gives:

$$\begin{array}{l} \text{T}=\text{Y}-\mu =\left(\text{I}-\text{Z}{\left({\text{Z}}^{\text{t}}{\text{H}}^{-1}\text{Z}\right)}^{-1}{\text{Z}}^{\text{t}}{\text{H}}^{-1}-{\hat{\sigma }}_{b}^{2}\Phi \text{P}\right)\text{Y}, \end{array}$$

where $\text{H}={\hat{\sigma }}_{b}^{2}\Phi +{\hat{\sigma }}_{e}^{2}{\text{I}}_{N}$, and **P = H**^**−1**^ − **H**^**−1**^**Z**(**Z**^**t**^**H**^**−1**^**Z**) ^**−1**^**Z**^**t**^**H**^**−1**^. For a dichotomous phenotype, use of the generalized linear mixed model might be considered an appropriate approach, but we estimated **T** in the same way for quantitative phenotypes when individuals were randomly selected because of its superior statistical power (Won and Lange, 2013).

### Score for Quasi-Likelihood

We let **1** *w* be the *w* × 1 column vector, of which the elements are one. The score based on quasi-likelihood for variant *m* in study *k* is defined by:

$$\begin{array}{l} u_{m,k}={\text{T}}_{k}^{t}\left({\text{I}}_{N_{k}}-1_{N_{k}}{\left(1_{N_{k}}^{t}\Phi_{k}^{-1}1_{N_{k}}\right)}^{-1}1_{N_{k}}^{t}\Phi_{k}^{-1}\right){\text{X}}_{k}^{m}. \end{array}$$

If we denote the covariance between *x*_*m, k*_ and $x_{m^{\prime },k}$ by $\sigma_{mm^{\prime },k}$, then $cov\left({\text{X}}_{k}^{m},{\text{X}}_{k}^{m^{\prime }}\right)=\sigma_{mm^{\prime },k}\Phi_{k}$, and $\sigma_{mm^{\prime },k}$ is estimated by the empirical covariance. We let:

$$\begin{array}{l} \Sigma_{k}=\left[\begin{array}{ccc} \sigma_{11,k} & \ldots & \sigma_{1M,k}\\ \vdots & \ddots & \vdots \\ \sigma_{M1,k} & \ldots & \sigma_{MM,k} \end{array}\right]. \end{array}$$

If we let ${\text{H}}_{k}=\Phi_{k}-1_{N_{k}}{\left({1_{N_{k}}^{t}\Phi_{k}^{-1}1}_{N_{k}}\right)}^{-1}1_{N_{k}}^{t}$, the variance-covariance matrix of u_*m, k*_(Choi et al., 2014) was shown to be:

$$\begin{array}{l} var\left[\begin{array}{c} {\text{T}}_{k}^{t}\left({\text{X}}_{k}^{1}-\hat{E}\left({\text{X}}_{k}^{1}\right)\right)\\ \vdots \\ {\text{T}}_{k}^{t}\left({\text{X}}_{k}^{M}-\hat{E}\left({\text{X}}_{k}^{M}\right)\right) \end{array}\right]=\left({\text{T}}_{k}^{t}{\text{H}}_{k}{\text{T}}_{k}\right)\Sigma_{k}. \end{array}$$

The score vector of rare variants in study *k* can be defined by:

$$\begin{array}{l} {\text{U}}_{k}=\frac{1}{\sqrt{{\text{T}}_{k}^{t}{\text{H}}_{k}{\text{T}}_{k}}}{\text{T}}_{k}^{t}\left({\text{I}}_{N_{k}}-1_{N_{k}}{\left({1_{N_{k}}^{t}\Phi_{k}^{-1}1}_{N_{k}}\right)}^{-1}1_{N_{k}}^{t}\Phi_{k}^{-1}\;\right){\text{X}}_{k}. \end{array}$$

The score statistic tests whether the coded genotypes are linearly independent from the phenotypes; for dichotomous phenotypes, it is equivalent to comparing the minor allele frequencies (MAFs) between cases and controls.

### Homogeneous Model

The homogeneous model assumes that the effect sizes of each variant are expected to be similar among different studies, and thus the proposed scores for each study can be collapsed across studies as follows:

$$\begin{array}{l} {\text{U}}^{Hom}\equiv \underset{k}{\sum }v_{k}{\text{U}}_{k}^{t},\;\Sigma^{Hom}\equiv var\left({\text{U}}^{Hom}\right)=\;\underset{k}{\sum }v_{k}^{2}\Sigma_{k}. \end{array}$$

Here, we set *v*_*k*_ to be one. However, the proposed statistics are sometimes unavailable, and the appropriate choice can vary according to the available information. For instance, if standardized test statistics and sample sizes are available, then the inverse function to the square root of the sample size can be utilized.

Rare variant association analysis can be categorized into burden and variance-component tests (Li and Leal, 2008; Price et al., 2010; Neale et al., 2011; Wu et al., 2011). The burden test is known to be the most powerful if all rare variants have either deleterious or protective effects on disease; otherwise, the variance-component test is more efficient (Neale et al., 2011). If we let $\chi_{1}^{2}$ be a chi-square distribution with a single degree of freedom, the burden test for a homogeneous model becomes:

$$S_{burden}^{Hom}=\frac{{\left(U^{Hom}\right)}^{t}W_{W}1_{M}1_{M}^{t}W_{W}U^{Hom}}{1_{M}^{t}W_{W}\Sigma^{Hom}W_{W}1_{M}}\sim \chi_{1}^{2}\text{ under }H_{0}.$$

Variance component tests use the collapsed squared scores (Neale et al., 2011; Wu et al., 2011) and can be expressed by:

$$S_{SKAT}^{Hom}={\left(U^{Hom}\right)}^{t}W_{W}I_{M}W_{W}U^{Hom}.$$

We denote eigenvalues for ${\left(\Sigma^{Hom}\right)}^{1/2}{\text{W}}_{W}{\text{W}}_{W}{\left(\Sigma^{Hom}\right)}^{1/2}$ by λ_*l*_. If we let $\chi_{1,l}^{2}$ be an independent chi-square distribution with a single degree of freedom, the variance component test for the homogeneous model follows:

$$\begin{array}{l} S_{SKAT}^{Hom}\sim \overset{M}{\underset{l=1}{\sum }}\lambda_{l}\chi_{1,l}^{2}\text{ under }H_{0}. \end{array}$$

A balanced approach for both scenarios can be achieved by the SKAT-O type statistic (Lee et al., 2012a). For a certain *c* between 0 and 1, we consider:

$${\left(U^{Hom}\right)}^{t}W_{W}\left(\left(1-c\right)I_{M}+c1_{M}1_{M}^{t}\right)W_{W}U^{Hom}.$$

If we let its *p*-value be $pS_{c}^{Hom}$, the SKAT-O type statistic for *c*_0_ = 0 < *c*_1_ < … < *c_L_* = 1 is defined by:

$$\begin{array}{l} S_{SKATO}^{Hom}=p_{\min}^{Hom}=\text{min }\left\{pS_{0}^{Hom},pS_{0.01}^{Hom},pS_{0.04}^{Hom},pS_{0.09}^{Hom},pS_{0.16}^{Hom},pS_{0.25}^{Hom},pS_{1}^{Hom}\right\}. \end{array}$$

Its *p*-value can be calculated with the numerical algorithm for the FARVAT statistic (Choi et al., 2014).

Last, rare variant association analysis utilizes rare variants, but the definition of a rare variant is not clear. VT approaches are very useful in such scenarios. We assume that rare variants are sorted in ascending order of overall MAF. We let **1**_(m)_ be an *M*-dimensional column vector whose 1st,…, *m*th elements are 1 and the others are 0. If we let:

$$\begin{array}{l} U_{\left(m\right)}^{Hom}=\overset{K}{\underset{k=1}{\sum }}v_{k}1_{\left(m\right)}^{t}{\text{W}}_{W}{\text{U}}_{k}^{t}=1_{\left(m\;\right)}^{t}{\text{W}}_{W}{\text{U}}^{Hom}, \end{array}$$

then the covariance between $U_{\left(m\right)}^{Hom}$ and $U_{\left(m^{\prime }\right)}^{Hom}$is:

$$\begin{array}{l} 1_{\left(m\right)}^{t}{\text{W}}_{W}\Sigma^{Hom}{\text{W}}_{W}1_{\left(m^{\prime }\right)}. \end{array}$$

Therefore, we let:

$$\begin{array}{l} T_{\left(m\right)}^{Hom}=\frac{U_{\left(m\right)}^{Hom}}{\sqrt{1_{\left(m\right)}^{t}{\text{W}}_{W}\Sigma^{Hom}{\text{W}}_{W}1_{\left(m\right)}}}. \end{array}$$

If we denote the realization of $T_{\left(m\right)}^{Hom}$ by *t*_(m)_ and let *t*_(|max|)_ = max{ |*t*_(1)_|,…, |*t*_(M)_|}, the *p*-value for the VT method can be calculated by:

$$\begin{array}{l} 1-P\left(|T_{\left(1\right)}^{Hom}|>t_{\left(\left|\max\right|\right)},\cdots \;,|T_{\left(M\right)}^{Hom}|>t_{\left(\left|max\right|\right)}\right). \end{array}$$

Here, ${\left({\text{T}}_{\left(1\right)}^{Hom},\cdots ,{\text{T}}_{\left(\text{M}\right)}^{Hom}\right)}^{t}$ follows the multivariate normal distribution with mean 0 and the following correlation matrix:

$$\Psi^{Hom}={\left(\Psi_{mm^{\prime }}^{Hom}\right)}_{M\times M},$$

where $\Psi_{mm^{\prime }}^{Hom}=\frac{1_{\left(m\right)}^{t}W_{W}\Sigma^{Hom}W_{W}1_{\left(m^{\prime }\right)}}{\sqrt{\left(1_{\left(m\right)}^{t}W_{W}\Sigma^{Hom}W_{W}1_{\left(m\right)}\right)\left(1_{\left(m^{\prime }\right)}^{t}W_{W}\Sigma^{Hom}W_{W}1_{m^{\prime }}\right)}}.$

### Heterogeneous Model

As in the homogeneous model, we propose burden and variance component tests for the heterogeneous model. The heterogeneous model assumes that the effects of specific variants are heterogeneous among studies. If we let *E*(*u*_*m, k*_) = β_*mk*_, the null hypothesis can be expressed by β_11_ = … = β_*MK*_ = 0, and we consider the following score vector and its variance matrix:

$$\begin{array}{l} {\text{U}}^{Het}\equiv {\left(\begin{array}{c} v_{1}{\text{U}}_{1}\ldots v_{K}{\text{U}}_{K} \end{array}\right)}^{t},\;\Sigma^{Het}\equiv var\;\left(vec\left(\text{U}\right)\right)\\ \;=\underset{k}{\sum }\left(v_{k}^{2}\Sigma_{k}⊗{\text{e}}_{kk}\;\right), \end{array}$$

where **e**_*kk*_ is a *K* × *K* dimensional matrix whose (*k, k*) element is 1 and the others are 0. Then, the burden test can be expressed as:

$$\begin{array}{l} S_{burden}^{Het}=\frac{{\text{U}}^{Het}\left({\text{W}}_{W}⊗{\text{W}}_{B}\right)1_{MK}1_{MK}^{t}\left({\text{W}}_{W}⊗{\text{W}}_{B}\right){{\text{U}}^{Het}}^{t}}{1_{MK}^{t}\left({\text{W}}_{W}⊗{\text{W}}_{B}\right)\Sigma^{Het}\left({\text{W}}_{W}⊗{\text{W}}_{B}\right)1_{MK}}\sim \chi_{1}^{2}\text{ under}H_{0}. \end{array}$$

We let:

$$\begin{array}{l} {\text{R}}_{c}^{Het}=\left(1-c\right){\text{I}}_{MK}{+c1}_{MK}1_{MK}^{t},\;S_{c}^{Het}\\ \;={\text{U}}^{Het}\left({\text{W}}_{W}⊗{\text{W}}_{B}\right){\text{R}}_{c}^{Het}\left({\text{W}}_{W}⊗{\text{W}}_{B}\right){{\text{U}}^{Het}}^{t}, \end{array}$$

and we let $\left(\lambda_{1}^{c},\ldots ,\lambda_{MK}^{c}\right)$ be the eigenvalues of:

$$\begin{array}{l} \underset{k}{\sum }\left(\Sigma_{k}⊗{\text{e}}_{kk}\right)\left({\text{W}}_{W}⊗{\text{W}}_{B}\right){\text{I}}_{MK}\left({\text{W}}_{W}⊗{\text{W}}_{B}\right)\underset{k}{\sum }\left(\Sigma_{k}⊗{\text{e}}_{kk}\right). \end{array}$$

Then, $S_{c}^{Het}$ follows:

$$\begin{array}{l} S_{c}^{Het}\sim \;\overset{MK}{\underset{l=1}{\sum }}\lambda_{l}^{c}\chi_{1,l}^{2}\text{ under }H_{0}. \end{array}$$

Therefore, the variance component test is defined by:

$$\begin{array}{l} S_{SKAT}^{Het}={\text{U}}^{Het}\left({\text{W}}_{W}⊗{\text{W}}_{B}\right){\text{I}}_{MK}\left({\text{W}}_{W}⊗{\text{W}}_{B}\right){{\text{U}}^{Het}}^{t}\\ \;=S_{0}^{Het}\sim \;\overset{MK}{\underset{l=1}{\sum }}\lambda_{l}^{0}\chi_{1,l}^{2}\text{ under }H_{0}. \end{array}$$

If we denote the *p*-value for $S_{c}^{Het}$ by *p*$S_{c}^{Het}$, the SKAT-O-type statistic is defined by:

$$\begin{array}{l} S_{SKATO}^{Het}=p_{\min}^{Het}=min\;\left\{pS_{0}^{Het},pS_{0.01}^{Het},pS_{0.04}^{Het},pS_{0.09}^{Het},pS_{0.16}^{Het},pS_{0.25}^{Het},pS_{1}^{Het}\right\}, \end{array}$$

and its *p*-value is also obtained by the numerical algorithm for the FARVAT statistic (Choi et al., 2014).

### Simulation Model

The performance of metaFARVAT was evaluated via extensive simulation studies. metaFARVAT can be applied to population-based and case-control designs by calculating GRM among samples. Therefore, we only focused on family-based designs in our simulation studies and considered unbalanced families consisting of trios, nuclear families, and extended families with three generations; the family structures that we considered are presented in Supplementary Figure 1. The families for our simulations were randomly selected from these different family structures. To generate rare variants, 1,200 haplotypes with 50,000 base pairs were generated under a coalescent model using the software COSI (Schaffner et al., 2005). Each haplotype was generated by setting the mutation rate to 1.5 × 10^−8^, and haplotypes were randomly chosen with replacement to build founder genotypes. We defined variants with MAFs < 0.01 as being rare, and 60 rare variants were randomly selected from their haplotypes. Then, non-founder haplotypes were chosen from their parents' haplotypes in Mendelian fashion under the assumption of no recombination.

Phenotypes were generated under the null and alternative hypotheses. Simulation of dichotomous phenotypes was performed using the liability threshold model. Once the quantitative phenotypes were generated, they were transformed into case-control status for dichotomous phenotypes. If quantitative phenotypes were larger than the threshold, they were considered affected and otherwise were considered unaffected. The threshold was chosen to preserve the assumed disease prevalence of 0.1. If the disease prevalence is misspecified, loss of statistical power is expected; however, it has been shown with simulation studies that the effect of misspecification is not very substantial (Won and Lange, 2013). To allow for the ascertainment bias of dichotomous phenotypes in our simulation studies, we assumed that families with at least one affected subject were selected for analysis.

Quantitative phenotypes were defined by summing the phenotypic mean, genetic effect, polygenic effect, main genetic effect, and random error, and we assumed there was no environmental effect shared between family members. The phenotypic mean was denoted by α = 0.3. The polygenic effect for each founder was independently generated from *N*(0, σ_*g*_^2^ = 1), and for non-founders, the average of maternal and paternal polygenic effects was combined with values independently sampled from *N*(0, 0.5σ_*g*_^2^). Random error was independently sampled from *N*(0, σ_*e*_^2^ = 1). Therefore, the heritability of the simulated trait is 0.5. The genetic effect at variant *m* in study *k* was the product of β_*mk*_ and the number of disease susceptibility alleles. To evaluate the type-1 error estimates, β_*mk*_ was assumed to be 0. To evaluate the statistical power estimates, if we let *h_a_*^2^ be the proportion of variance explained by rare variants, β_*mk*_ values were iteratively sampled with a two-step approach. $\beta_{mk}^{\left(0\right)}$ were first sampled from *U*(0,1). Then, if we let:

$$\begin{array}{l} \nu_{k}=\sqrt{\frac{\left(\sigma_{g}^{2}+\sigma_{e}^{2}\right)h_{a}^{2}}{\left(1-h_{a}^{2}\right)\sum_{m=1}^{M}\left[{\left(\beta_{mk}^{\left(0\right)}\right)}^{2}2p_{m}\left(1-p_{m}\;\right)\right]}}, \end{array}$$

β_*mk*_ values were sampled from the uniform distribution *U*(0, *v_k_*). This procedure was repeated until *v_k_* converged. We assumed that *h_a_*^2^ = 0.01. β_*mk*_ was generated from heterogeneous or homogeneous scenarios. For homogeneous scenarios, we assumed that the effects of each rare variant were in the same direction in all studies. For heterogeneous scenarios, the signs (±) of β_*mk*_ values sampled from *U*(*0*,*v_k_*) were chosen randomly.

### Application to COPD Data

We considered previously reported WES data from Boston Early-Onset COPD Study (EOCOPD) families and COPDGene case-control subjects for meta-analysis (Qiao et al., 2016). Details of the EOCOPD study have been described previously (Silverman et al., 1998). The EOCOPD data are derived from an extended pedigree-based design. Probands were 53 years old or younger with prebronchodilator forced expiratory volume in 1 s (FEV_1_) of ≤40%, physician-diagnosed COPD, and without severe alpha-1 antitrypsin deficiency. All first-degree relatives, older second-degree relatives, and additional affected family members were enrolled. There were 49 pedigrees with at least two affected family members selected for WES. COPDGene was a multi-center study of smokers with and without COPD and included African-Americans and non-Hispanic whites (Regan et al., 2010). The COPDGene participants, consisting of 10,192 smokers, had at least 10 pack years of smoking, and their ages were between 45 and 80 years. From the COPDGene study, 204 COPD subjects with GOLD (Global Initiative for Chronic Obstructive Lung Disease) spirometry grades 3–4 (post-bronchodilator FEV_1_ <50% and ratio of FEV_1_ to forced vital capacity (FEV_1_/FVC) <0.7), as well as 195 controls with normal spirometry (frequency-matched to COPD cases on pack-years of cigarette smoking), were chosen for WES.

Sequencing for both cohorts was performed at the University of Washington (Seattle, WA), using Nimblegen V2 capture (Roche NimbleGen, Inc., Madison, WI), and the Illumina platform (Illumina, Inc., San Diego, CA). Participants selected from the COPDGene cohort were sequenced via the NHLBI Exome Sequencing Program, and EOCOPD subjects were sequenced as part of the Center for Mendelian Genomics. Quality control (QC) filtering for both data sets was performed by the method of Qiao et al. (2016) and filtered out variants with Mendelian errors (for family-based data), call rate <99%, Hardy-Weinberg equilibrium *p*-value <10^−8^, and average sequencing depth <12, as well as excluding subjects with pedigree, racial, or sex mismatches. After QC, there were 303 individuals from 49 families and 124,288 variants in the EOCOPD data set, and there were 394 unrelated individuals and 108,443 variants in the COPDGene data set. For rare variant analyses, we assumed that variants with MAFs <5% in dbSNP were rare, and in both studies, we separately filtered out singleton variants or genes with minor allele counts (MACs) <10. Finally, 88,737 rare variants in 13,935 genes were analyzed in the EOCOPD data set, and 24,846 rare variants in 10,550 genes were tested in the COPDGene data set. For both EOCOPD and COPDGene data, GRMs were estimated for variants with MAFs >5% and were incorporated as variance-covariance matrices of genotypes to adjust for population substructure. Effects of covariates for binary phenotypes were adjusted by using the BLUP as an offset. First, we fitted the linear mixed model with adjustments for age, sex, and pack-years of smoking as covariates, and then BLUP was set as the offset for the proposed methods. A description of the two datasets is provided in Supplementary Table 4.

## Results

### Evaluation of metaFARVAT With Simulated Data

To evaluate statistical validity, type-1 error estimates for both dichotomous and quantitative phenotypes were calculated at various significance levels using 20,000 replicates of 200 unbalanced families. For each replicate, we performed three different meta-analyses, including 3, 6, and 9 studies. Table 1 shows empirical type-1 error estimates for homogeneous metaFARVAT (metaFARVAT^Hom^) and heterogeneous metaFARVAT (metaFARVAT^Het^) at the 0.1, 0.01, 10^−3^, and 10^−4^ significance levels with dichotomous phenotypes. Estimates of type-1 error rates were virtually equal to nominal significance levels. However, VT type metaFARVAT^Hom^ showed inflation, especially when there were three studies, and if the number of rare variants is small, it is not recommended. Quantile-quantile (QQ) plots in Supplementary Figures 2–4 also show consistent results. Therefore, we conclude that the proposed metaFARVAT^Hom^ and metaFARVAT^Het^ are statistically valid.

**Table 1:** Type-1 error estimates from simulation study with dichotomous phenotypes.

			**Dichotomous**			
	**# Studies**	**Significance level**	**Burden**	**SKAT**	**SKAT-O**	**VT**
Hom	3	0.1	0.0960	0.0950	0.0953	0.1100
		0.01	0.0103	0.0099	0.0100	0.0116
		10^−3^	0.0009	0.0012	0.0014	0.0017
		10^−4^	0.0001	0.0001	0.0001	0.0004
	6	0.1	0.1002	0.0953	0.0957	0.1018
		0.01	0.0094	0.0085	0.0088	0.0106
		10^−3^	0.0008	0.0009	0.0008	0.0011
		10^−4^	0.0001	0.0000	0.0000	0.0001
	9	0.1	0.1000	0.1015	0.1025	0.1018
		0.01	0.0096	0.0098	0.0093	0.0110
		10^−3^	0.0007	0.0009	0.0007	0.0015
		10^−4^	0.0001	0.0000	0.0000	0.0001
Het	3	0.1	0.0987	0.1006	0.0981	–
		0.01	0.0100	0.0091	0.0094	–
		10^−3^	0.0008	0.0008	0.0013	–
		10^−4^	0.0001	0.0002	0.0002	–
	6	0.1	0.1036	0.0986	0.0985	–
		0.01	0.0094	0.0106	0.0105	–
		10^−3^	0.0008	0.0014	0.0012	–
		10^−4^	0.0001	0.0003	0.0002	–
	9	0.1	0.1041	0.1026	0.1046	–
		0.01	0.0107	0.0095	0.0107	–
		10^−3^	0.0009	0.0011	0.0009	–
		10^−4^	0.0001	0.0002	0.0001	–

*The empirical type-1 error was estimated for the proposed methods with 20,000 replicates at the 0.1, 0.01, 10^−3^, and 10^−4^ significance levels for dichotomous phenotypes. We assumed that the number of rare variants is 60, and that their minor allele frequencies <0.01. Both homogeneous (Hom) and heterogeneous (Het) models were considered*.

Secondly, empirical power estimates for dichotomous phenotypes were calculated at the 2.5 × 10^−6^ significance level, showing the changes in power under different scenarios. Empirical power estimates were calculated with 2,000 replicates for seven different statistics: burden, SKAT, SKAT-O, and VT type statistics for metaFARVAT^Hom^ and burden, SKAT, and SKAT-O type statistics for metaFARVAT^Het^. Results are provided in Tables 2, 3 for homogeneous and heterogeneous scenarios, respectively. In addition, we compared the proposed methods with two meta-analysis methods based on the use of *p*-values across studies: the minimum *p*-value method and Fisher's method. If we let *p_k_* be the *p*-value from the *k*th study (*k* = 1,2,…,*K*), the minimum *p*-value and Fisher's method can be obtained by:

$$\begin{array}{l} \text{minP}=\text{min}\left(p_{k}\right)\sim \text{Beta}\left(1,K\right),\text{Fisher}=-2\overset{K}{\underset{k=1}{\sum }}\ln\;p_{k}\\ \;\sim \chi^{2}\left(df=2K\right)\text{ under}H_{0}. \end{array}$$

**Table 2:** Empirical power estimates for dichotomous phenotype for homogeneous variants among studies.

		**3 studies**				**6 studies**				**9 studies**			
**±**	**Method**	**SKAT**	**Burden**	**SKAT-O**	**VT**	**SKAT**	**Burden**	**SKAT-O**	**VT**	**SKAT**	**Burden**	**SKAT-O**	**VT**
60/0	Fisher	0.1990				0.6495				0.8940			
	minP	0.0315				0.0610				0.0715			
	Hom	0.0195	0.3590	0.3660	0.3915	0.1690	0.9265	0.9150	0.9240	0.4920	0.9975	0.9945	0.9965
	Het	0.0115	0.3390	0.4160	–	0.0750	0.9095	0.9330	–	0.1865	0.9930	0.9960	–
48/12	Fisher	0.0270				0.1060				0.2400			
	minP	0.0060				0.0070				0.0070			
	Hom	0.0105	0.0335	0.0670	0.0450	0.1105	0.2290	0.3720	0.2665	0.4000	0.5355	0.7565	0.5720
	Het	0.0045	0.0310	0.0720	–	0.0225	0.2080	0.3305	–	0.0760	0.4825	0.6325	–
30/30	Fisher	0.0000				0.0015				0.0035			
	minP	0.0000				0.0000				0.0000			
	Hom	0.0050	0.0000	0.0025	0.0010	0.0555	0.0000	0.0270	0.0000	0.2615	0.0000	0.1650	0.0065
	Het	0.0000	0.0000	0.0005	–	0.0020	0.0000	0.0015	–	0.0120	0.0000	0.0090	–
30/0	Fisher	0.0440				0.2090				0.4520			
	minP	0.0090				0.0170				0.0205			
	Hom	0.0140	0.0725	0.1145	0.0900	0.1790	0.4260	0.5760	0.4785	0.5515	0.7970	0.9125	0.8220
	Het	0.0070	0.0605	0.1290	–	0.0555	0.3905	0.5545	–	0.1410	0.7545	0.8590	–
24/6	Fisher	0.0075				0.0365				0.0895			
	minP	0.0020				0.0020				0.0015			
	Hom	0.0095	0.0045	0.0215	0.0085	0.1285	0.0465	0.1980	0.0610	0.4480	0.1440	0.5480	0.1765
	Het	0.0025	0.0035	0.0240	–	0.0225	0.0340	0.1215	–	0.0630	0.1105	0.2890	–
15/15	Fisher	0.0000				0.0030				0.0045			
	minP	0.0000				0.0010				0.0005			
	Hom	0.0020	0.0000	0.0005	0.0010	0.0550	0.0000	0.0270	0.0025	0.2700	0.0000	0.1650	0.0060
	Het	0.0000	0.0000	0.0000	–	0.0025	0.0000	0.0025	–	0.0090	0.0000	0.0030	–

*Empirical power of burden, SKAT, SKAT-O, and VT type of metaFARVAT^Hom^ and metaFARVAT^Het^ was calculated for dichotomous phenotypes with homogeneous effects at the 2.5 × 10^−6^ significance level*.

**Table 3:** Empirical power estimates for dichotomous phenotype for heterogeneous variants among studies.

		**3 studies**				**6 studies**				**9 studies**			
**±**	**Method**	**SKAT**	**Burden**	**SKAT-O**	**VT**	**SKAT**	**Burden**	**SKAT-O**	**VT**	**SKAT**	**Burden**	**SKAT-O**	**VT**
48/12	Fisher	0.0240				0.1040				0.2235			
	minP	0.0065				0.0070				0.0105			
	Hom	0.0040	0.0340	0.0425	0.0460	0.0130	0.2170	0.2450	0.2555	0.0420	0.5200	0.5305	0.5680
	Het	0.0080	0.0325	0.0590	–	0.0270	0.1865	0.3180	–	0.0715	0.4555	0.6115	–
30/30	Fisher	0.0000				0.0030				0.0020			
	minP	0.0000				0.0000				0.0000			
	Hom	0.0005	0.0000	0.0000	0.0005	0.0005	0.0000	0.0005	0.0000	0.0015	0.0000	0.0015	0.0000
	Het	0.0005	0.0000	0.0005	–	0.0050	0.0000	0.0030	–	0.0090	0.0000	0.0070	–
30/0	Fisher	0.0460				0.2220				0.4690			
	minP	0.0115				0.0145				0.0160			
	Hom	0.0065	0.0670	0.0945	0.0880	0.0400	0.4385	0.4595	0.4730	0.1185	0.7880	0.7875	0.7980
	Het	0.0060	0.0570	0.1340	–	0.0510	0.3930	0.5580	–	0.1370	0.7425	0.8380	–
24/6	Fisher	0.0095				0.0325				0.0850			
	minP	0.0030				0.0035				0.0030			
	Hom	0.0020	0.0070	0.0115	0.0120	0.0045	0.0470	0.0680	0.0655	0.0125	0.1520	0.1875	0.1900
	Het	0.0040	0.0065	0.0270	–	0.0215	0.0335	0.1230	–	0.0590	0.1185	0.2825	–
15/15	Fisher	0.0010				0.0015				0.0020			
	minP	0.0005				0.0010				0.0005			
	Hom	0.0000	0.0000	0.0000	0.0005	0.0000	0.0000	0.0000	0.0005	0.0000	0.0000	0.0000	0.0005
	Het	0.0015	0.0000	0.0015	–	0.0030	0.0000	0.0010	–	0.0095	0.0000	0.0060	–

*Empirical power of burden, SKAT, SKAT-O, and VT type of metaFARVAT^Hom^ and metaFARVAT^Het^ was calculated for dichotomous phenotypes with heterogeneous effects at the 2.5 × 10^−6^ significance level*.

According to our results, the minimum *p*-value approach usually performed the least efficiently, especially when there were equal numbers of protective and deleterious rare variants in the targeted gene. Moreover, the power of the minimum *p*-value approach was not much improved by including more studies in the meta-analysis. The Fisher approach always performed better than the minimum *p*-value approach but was less powerful than the metaFARVAT method, regardless of the scenario. Statistical power estimates depend on the scenarios, and both tables show that the best power estimates are usually found from metaFARVAT^Hom^ and metaFARVAT^Het^ under homogeneous and heterogeneous scenarios, respectively. Statistical power estimates also depend on the proportion of rare variants with deleterious or protective effects on the phenotypes, which is often unknown. For example, when all rare causal variants had deleterious effects on the phenotype, burden, and VT type metaFARVAT outperformed all other approaches, but if there were variants with deleterious and protective effects, SKAT-type metaFARVAT was the most efficient. SKAT-O metaFARVAT was not always the most powerful, but its empirical power estimates were usually very close to those of the most efficient approach.

The proposed methods can be applied to quantitative phenotypes, and results for quantitative phenotypes are provided in Supplementary Tables 1–3 and Supplementary Figures 5–7. For quantitative phenotypes, we compared our method with RAREMETAL and seqMeta, since these two methods can only be applied to quantitative phenotypes. Both approaches performed better than the proposed approach for homogeneous scenarios. However, RAREMETAL does not provide the SKAT-O type statistic and seqMeta does not provide the VT type statistic. seqMeta performed better than RAREMETAL in most scenarios and was similar to metaFARVAT^Hom^ under homogeneous scenarios. The SKAT-O type statistic in seqMeta did not perform well when there were as many protective variants as deleterious variants in the gene. metaFARVAT^Het^ outperformed other methods when the effects of each rare variant differed among studies and when there were variants with deleterious and protective effects within a gene.

### Application to COPD Data

To identify rare variants associated with COPD, we separately conducted rare variant analyses with EOCOPD and COPDGene data. Manhattan and QQ plots are provided in Supplementary Figure 8. According to the results, there were no exome-wide significant genes. We also conducted meta-analysis with metaFARVAT^Hom^ and metaFARVAT^Het^. For both statistics, *v*_1_ and *v*_2_ were set to 1. The QQ plots in Supplementary Figure 9 show that SKAT-O type metaFARVAT^Het^ and metaFARVAT^Hom^ preserved the nominal significance level. However, VT type metaFARVAT exhibited some inflation, and its results are therefore not included in Table 4. Manhattan plots are provided in Supplementary Figure 10. The Bonferroni-corrected 0.05 genome-wide significance level was 6.76 × 10^−6^ and is indicated by a solid blue line. Table 4 shows that *DLEC1* achieved genome-wide significance under both methods, and *ZNF441* was implicated with potentially significant results (*p*-value <10^−4^) by metaFARVAT^Hom^ SKAT-O.

**Table 4:** The candidate genes found by meta-analysis in COPD studies.

Method	Data	Gene	Sample size	Chromosome	Start	End	# Rare variants	MAC	P_B	P_S	*c*	P_O
metaFARVAT ^Hom^	EOCOPD &	*DLEC1*	697	3	38,080,978	38,163,785	9	66	1.21e-05	1.02e-04	0.25	5.24e-06
	COPDGene	*ZNF441*	697	19	11,890,983	11,892,255	2	24	9.88e-05	3.46e-04	1	2.13e-05
metaFARVAT ^Het^	EOCOPD & COPDGene	*DLEC1*	697	3	38,080,978	38,163,785	15	66	8.03e-06	9.54e-04	0.16	5.43e-06
FARVAT	EOCOPD	*DLEC1*	303	3	38,080,978	38,163,785	9	28	3.70e-03	0.147	1	7.24e-03
		*ZNF441*	303	19	11,890,983	11,892,255	2	13	1.12e-03	3.83e-03	1	1.37e-03
FARVAT	COPDGene	*DLEC1*	394	3	38,080,978	38,163,785	6	38	5.80e-04	7.96e-04	0.25	3.53e-04
		*ZNF441*	394	19	11,890,983	11,892,255	1	11	3.09e-02	3.09e-02	1	3.09e-02

*The definition of the acronyms in Table 4: (1) #rare variants, the number of rare variants in the gene; (2) MAC, minor allele counts; (3) P_B, the p-value of burden type test; (4) P_S, the p-value of SKAT type test; (5) c, the parameter used for SKAT-O; (6) P_O, the p-value of SKAT-O type test*.

## Discussion

Family-based association methods are robust against population substructure, and because of genetic homogeneity among family members, they are often utilized for rare variant association analyses. Multiple approaches have been proposed, and Tang and Lin (2015) provided a comprehensive overview of the statistical methods for meta-analysis of sequencing studies for discovering rare variant associations. According to their overview, RAREMETAL (Feng et al., 2014; Liu et al., 2014) and seqMeta (Chen et al., 2014) can be applied to family-based samples. However, these methods can consider only homogeneous effects with quantitative phenotypes, and no statistical methods for dichotomous phenotypes with family-based samples have been proposed.

In this study, we proposed a new meta-analysis method for family-based rare variant association analyses with dichotomous phenotypes, which can test both homogeneous and heterogeneous effects of variants in different studies. metaFARVAT can also be applied to quantitative phenotypes, and is able to combine all study designs, including family-based, case-control, and population-based designs. Furthermore, the proposed method was applied to a meta-analysis of EOCOPD and COPDGene data, and *DLEC1* was found to be genome-wide significant. *DLEC1* is a protein-coding gene encoding a cilia and flagella-associated protein. This gene has been implicated in several cancers but has not been previously associated with COPD. However, cilia-associated genes have been previously implicated in COPD (Tilley et al., 2015).

Despite the robustness and efficiency of the proposed method, there are still some limitations of the developed method. First, VT methods sort rare variants according to their MAFs and search the optimal threshold for rare variants. This approach is useful when it is not clear how to define rare variants. However, we found that type-1 error can be inflated if the number of rare variants is too small, and it is computationally intensive if there are a large number of variants to investigate. This problem can be solved by using a permutation method, and further investigation of this approach is necessary. Secondly, sufficiently large samples are necessary to guarantee that SKAT-O follows the assumed asymptotic distribution of the SKAT-O approach under the null hypothesis. Therefore, the SKAT-O type metaFARVAT also has this limitation when it is applied to a dichotomous phenotype with a small sample size. Thirdly, the proposed method cannot be applied to X- or Y-linked genes because the distributions of variants in X and Y chromosomes are different in males and females. Such an improvement will be considered in our future work. Lastly, in the simulation studies, we considered a limited number of rare variants and excluded noise variants. However, in practice, it is not known which rare variants are causal and which represent noise. Extensive simulations are thus necessary in our future work.

Despite the importance of rare variant analyses with family-based samples, this field of study has suffered over the last decades from a lack of statistical methods. In this study, we proposed new methods for family-based samples, enabling such statistical analyses.

## Availability and Implementation

The R package for metaFARVAT can be downloaded from http://healthstat.snu.ac.kr/software/metaFARVAT/.

## Author Contributions

LW and SW conceived and designed the experiments, performed the experiments, analyzed and interpreted the data, and drafted the manuscript. SL maintains the software homepage. DQ, MC, ES, and CL edited the manuscript. All authors read and approved the final version of the manuscript.

### Conflict of Interest Statement

In the past 3 years, ES received honoraria from Novartis for Continuing Medical Education Seminars and grant and travel support from GlaxoSmithKline. MC has received grant support from GSK. The remaining authors declare that the research was conducted in the absence of any commercial or financial relationships that could be construed as a potential conflict of interest.
