# Supplementary material for: metaFARVAT: An Efficient Tool for Meta-Analysis of Family-Based, Case-Control, and Population-Based Rare Variant Association Studies
Source: Front Genet. 2019 Jun 19;10:572. doi: 10.3389/fgene.2019.00572 (PMC6593391; doi:10.3389/fgene.2019.00572)
Supplement: Supplementary file 1 [file Data_Sheet_1.docx]

**Supplementary Figures, and Tables for “metaFARVAT: An Efficient Tool for Meta-analysis of Family-based Case-Control, and Population-Based Rare Variant Association Studies”**

Longfei Wang^1^, Dandi Qiao^2,3^, Michael Cho^2,3^, Edwin K Silverman^2,3^,

Christoph Lange^2,4^, Sungho Won^1, 5, 6*^

^1^Interdisciplinary Program in bioinformatics, Seoul National University, Seoul, 151-742, Korea

^2^Channing Division of Network Medicine, Department of Medicine, Brigham and Women’s Hospital and Harvard Medical School, Boston, MA 02115, USA

^3^Division of Pulmonary and Critical Care Medicine, Brigham and Women's Hospital, Boston, MA 02115, USA

^4^Department of Biostatistics, Harvard T.H. Chan School of Public Health, Boston, USA

^5^Department of Public Health Sciences, Seoul National University, Seoul, 151-742, Korea

^6^Institute of Health and Environment, Seoul National University, Seoul, Korea.

**Supplementary Figure 1: Family structures with different family members**.

| 1. $\mathrm{Trio}$ | 1. $Nuclear family with 4 members$ |
| --- | --- |
| **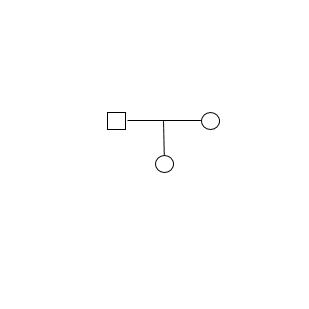** | **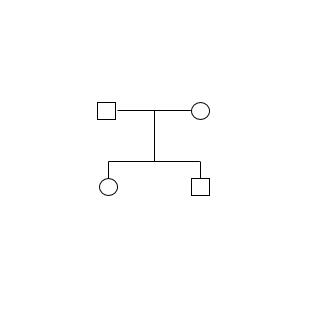** |
| 1. $Nuclear family with 5 members$ | 1. $Nuclear family with 6 members$ |
| 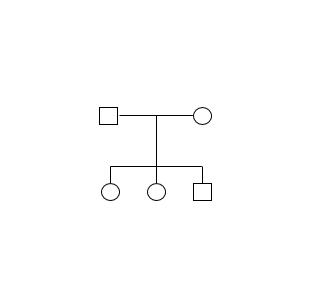 | 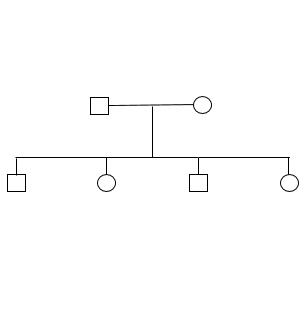 |
| 1. $Extended family with 7 members$ | 1. $Extended family with 8 members$ |
| 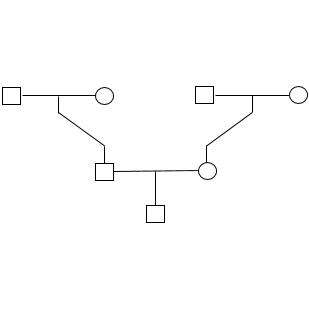 | 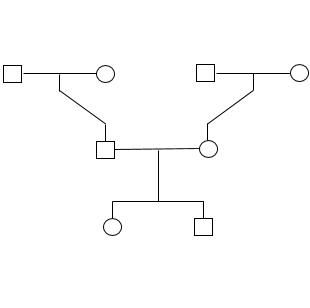 |
| 1. $Extended family with 9 members$ | 1. $Extended family with 10 members$ |
| 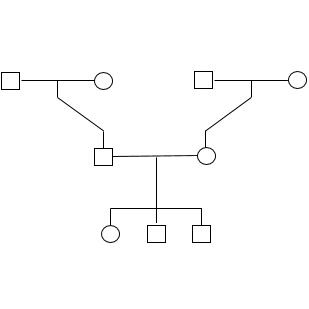 | 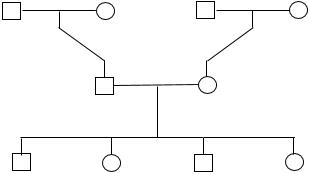 |

**Supplementary Figure 2: QQ plots for meta analyses of dichotomous phenotype based on 3 studies.** QQ plots were provided for results from the proposed methods under the null hypothesis. The empirical p-values were calculated under the null hypothesis with 20,000 replicates.

| 1. Burden-type metaFARVAT^Hom^ | 1. SKAT-type metaFARVAT^Hom^ |
| --- | --- |
| **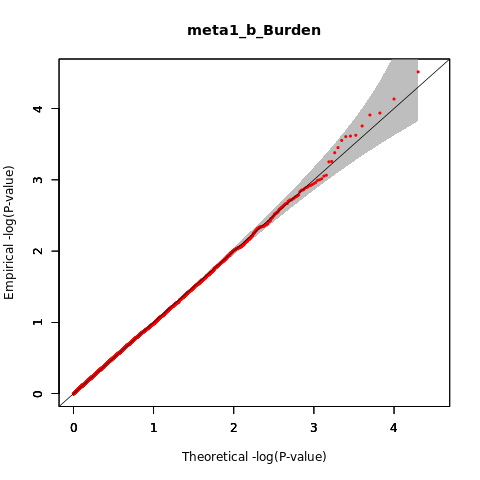** | **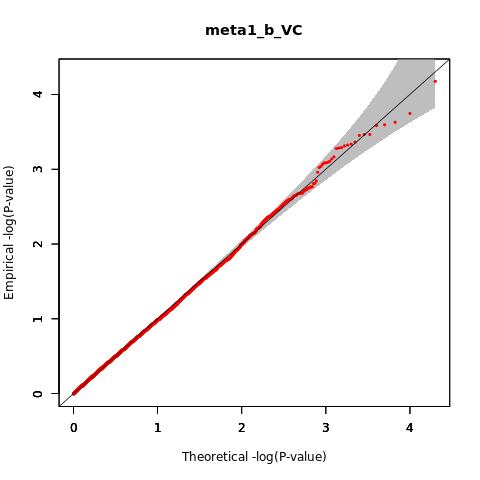** |
| 1. SKATO-type metaFARVAT^Hom^ | 1. VT-type metaFARVAT^Hom^ |
| 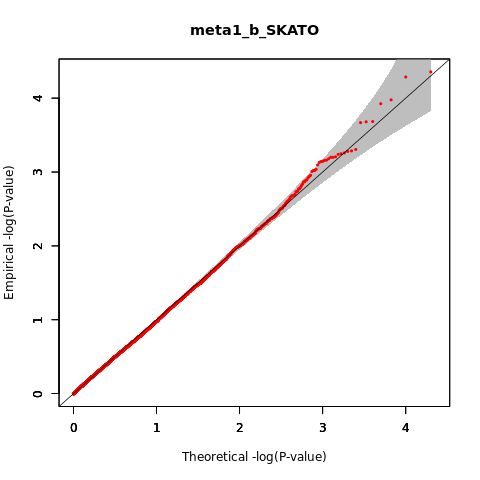 | 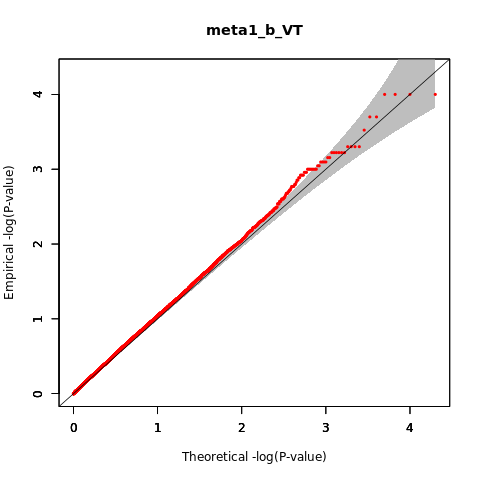 |
| 1. Burden-type metaFARVAT^Het^ | 1. SKAT-type metaFARVAT^Het^ |
| **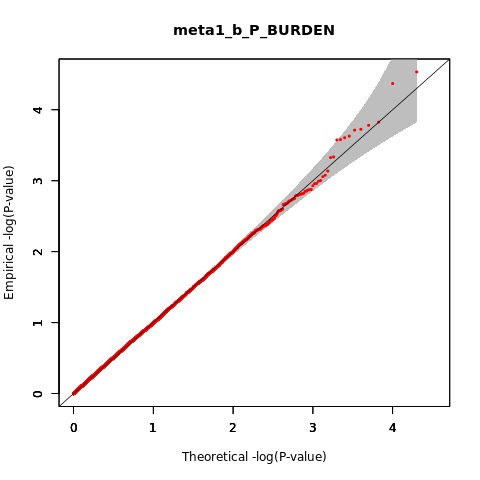** | **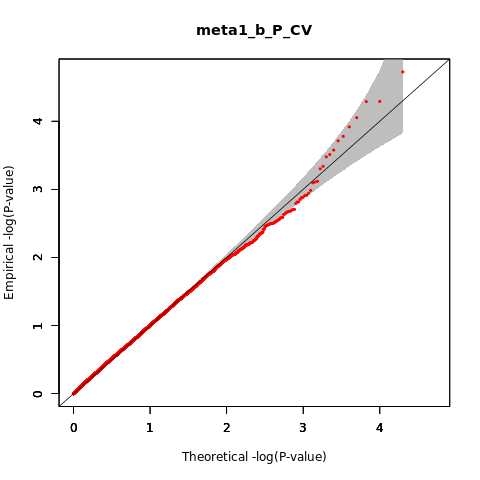** |
| 1. SKATO-type metaFARVAT^Het^ |  |
| 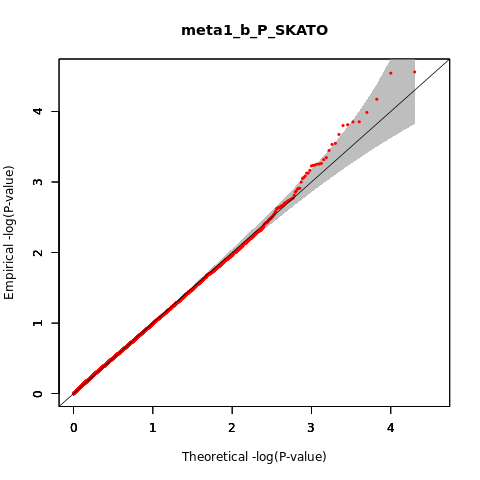 |  |

**Supplementary Figure 3: QQ plots for meta analyses of dichotomous phenotype based on 6 studies** QQ plots were provided for results from the proposed methods under the null hypothesis. The empirical p-values were calculated under the null hypothesis with 20,000 replicates.

| 1. Burden-type metaFARVAT^Hom^ | 1. SKAT-type metaFARVAT^Hom^ |
| --- | --- |
| **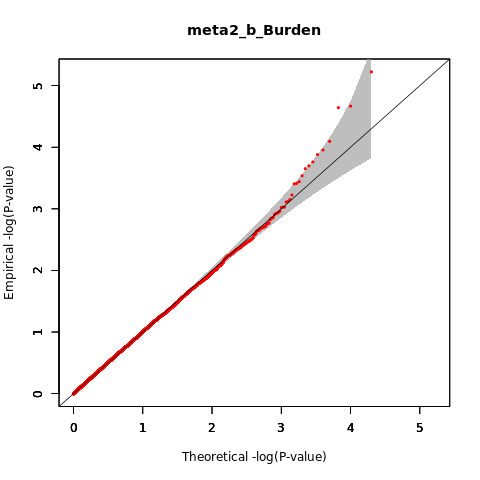** | **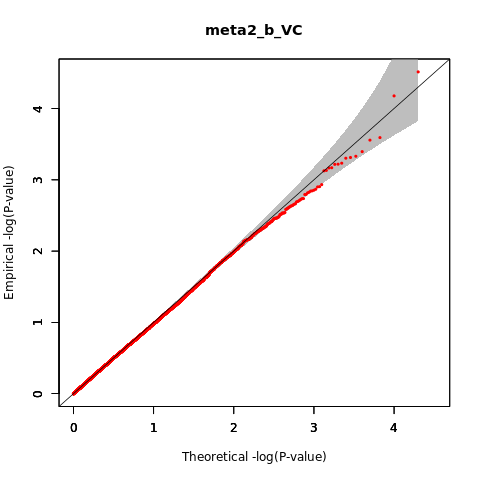** |
| 1. SKATO-type metaFARVAT^Hom^ | 1. VT-type metaFARVAT^Hom^ |
| 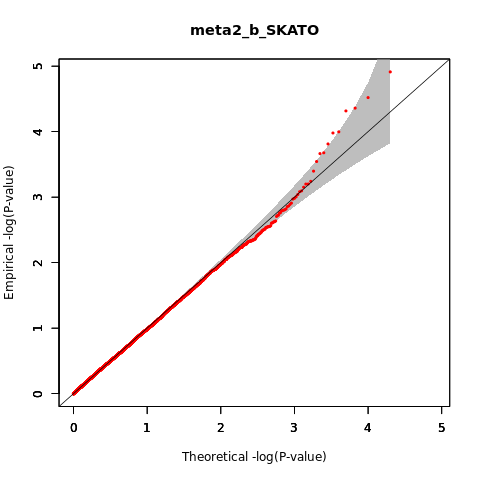 | 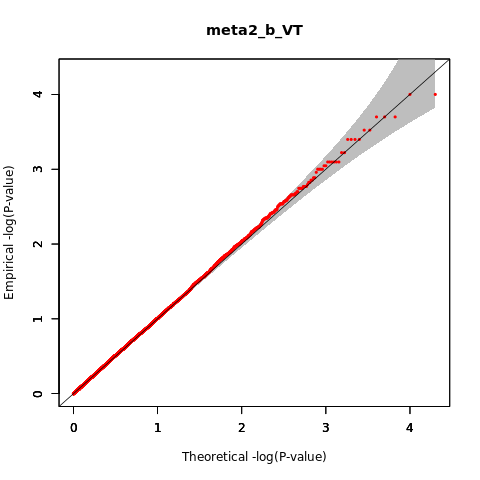 |
| 1. Burden-type metaFARVAT^Het^ | 1. SKAT-type metaFARVAT^Het^ |
| **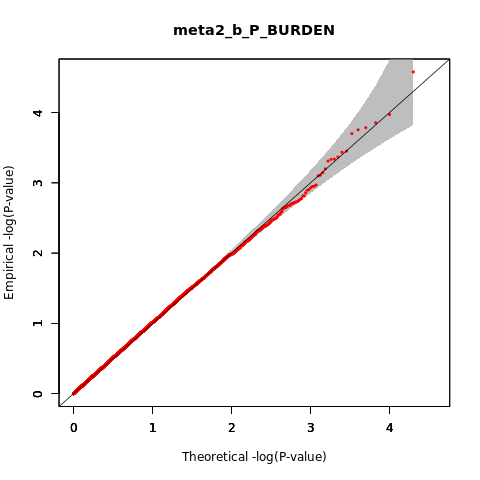** | **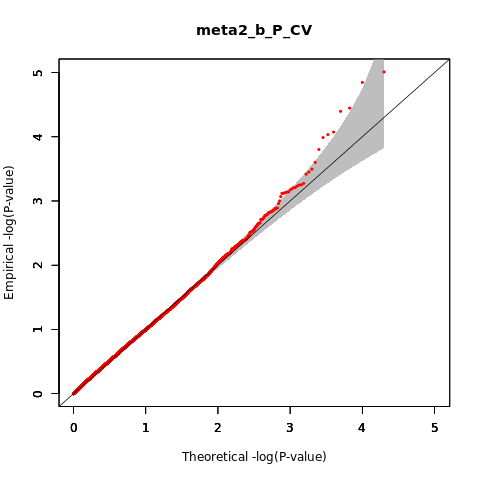** |
| 1. SKATO-type metaFARVAT^Het^ |  |
| 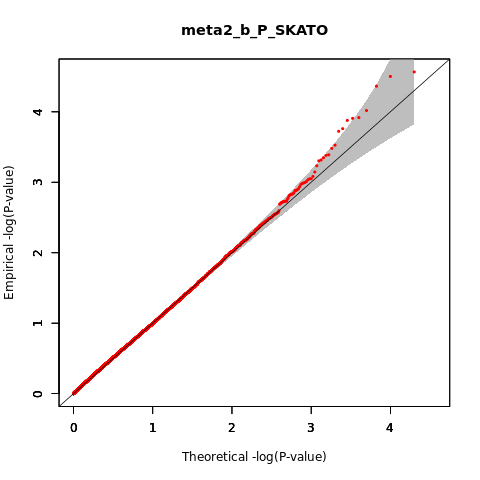 |  |

**Supplementary Figure 4: QQ plots for meta analyses of dichotomous phenotype based on 9 studies** QQ plots were provided for results from the proposed methods under the null hypothesis. The empirical p-values were calculated under the null hypothesis with 20,000 replicates.

| 1. Burden-type metaFARVAT^Hom^ | 1. SKAT-type metaFARVAT^Hom^ |
| --- | --- |
| **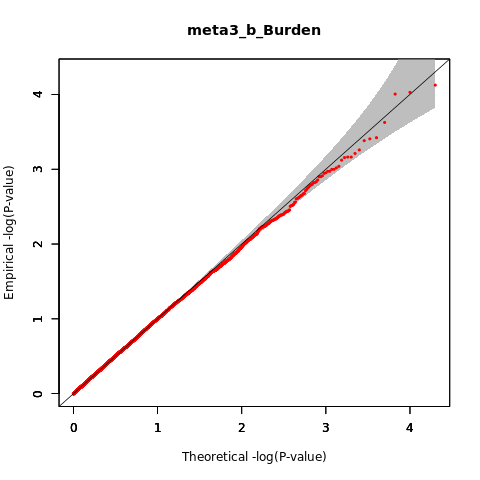** | **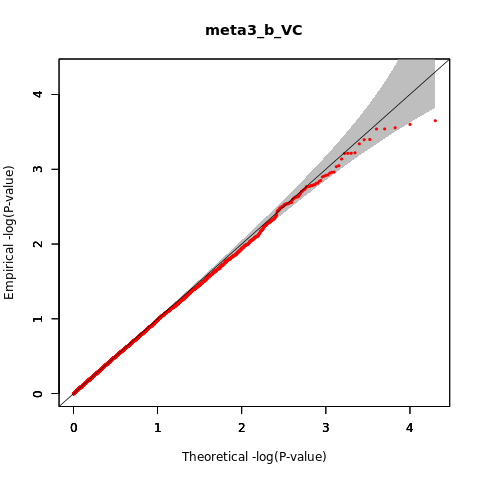** |
| 1. SKATO-type metaFARVAT^Hom^ | 1. VT-type metaFARVAT^Hom^ |
| 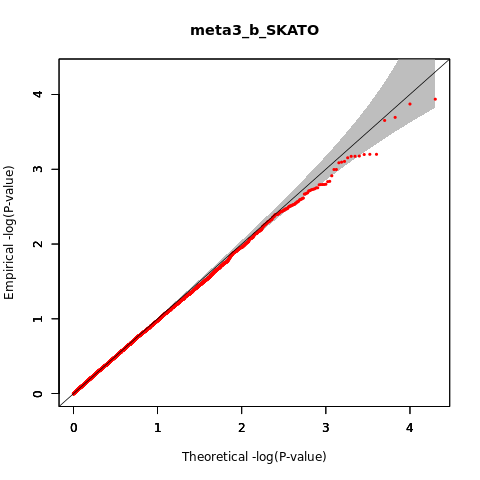 | 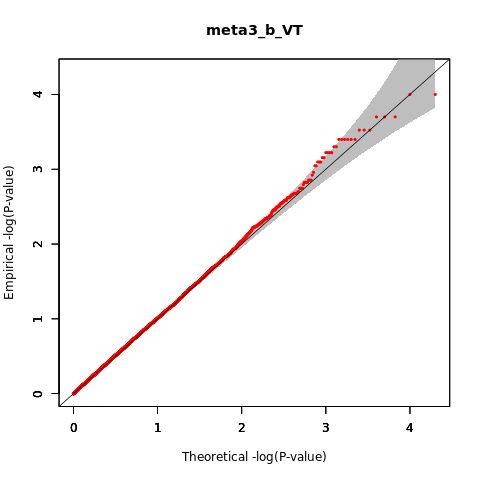 |
| 1. Burden-type metaFARVAT^Het^ | 1. SKAT-type metaFARVAT^Het^ |
| **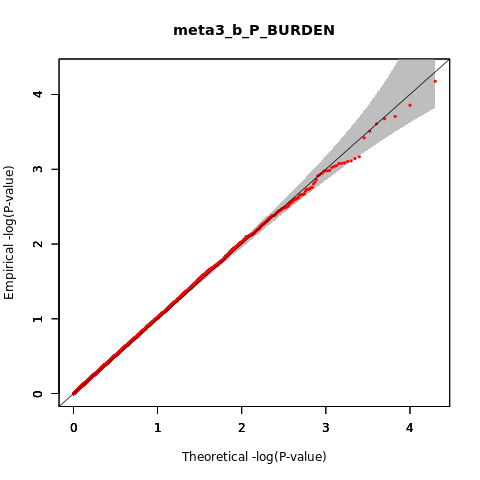** | **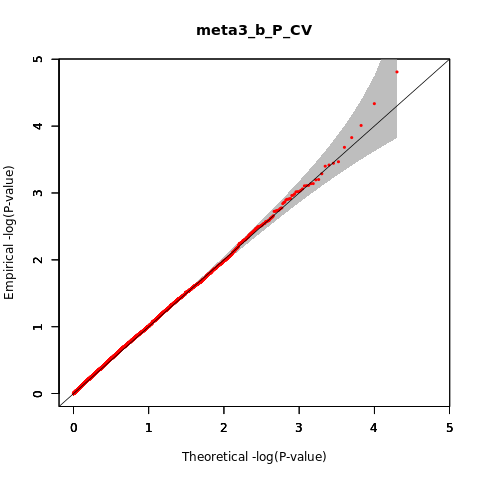** |
| 1. SKATO-type metaFARVAT^Het^ |  |
| 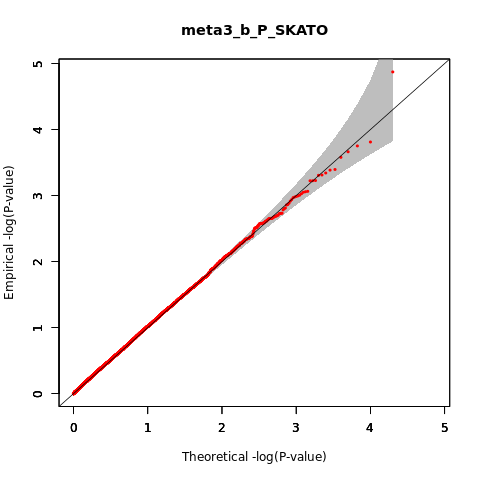 |  |

**Supplementary Figure 5: QQ plots for meta analyses of quantitative phenotype based on 3 studies** QQ plots were provided for results from the proposed methods under the null hypothesis. The empirical p-values were calculated under the null hypothesis with 20,000 replicates.

| 1. Burden-type metaFARVAT^Hom^ | 1. SKAT-type metaFARVAT^Hom^ |
| --- | --- |
| **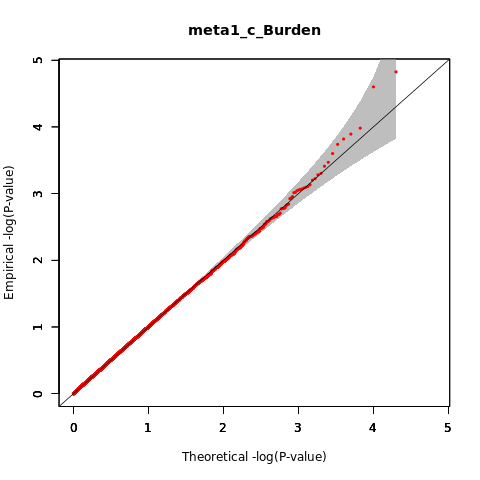** | **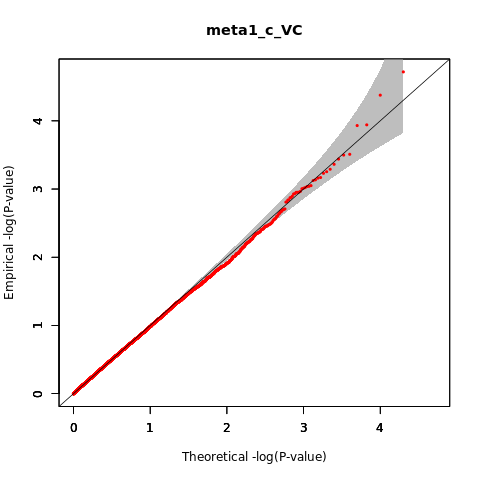** |
| 1. SKATO-type metaFARVAT^Hom^ | 1. VT-type metaFARVAT^Hom^ |
| 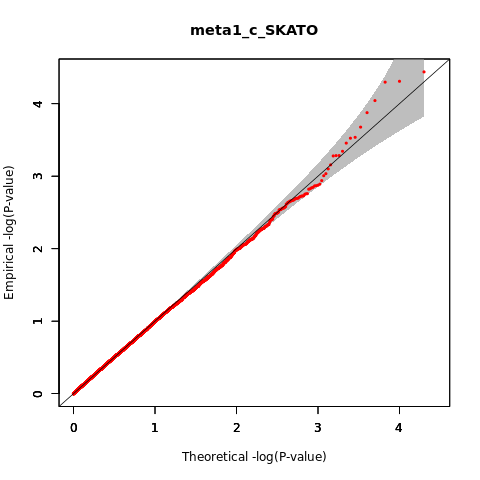 | 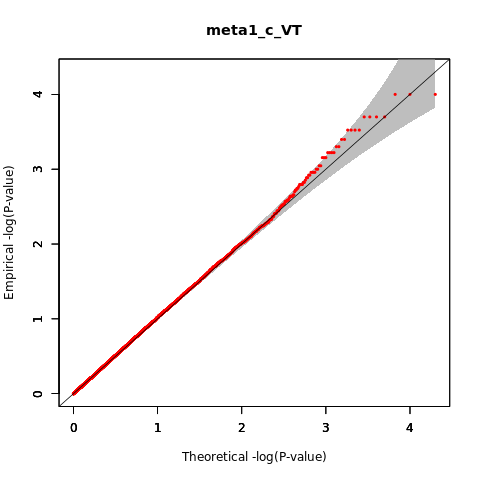 |
| 1. Burden-type metaFARVAT^Het^ | 1. SKAT-type metaFARVAT^Het^ |
| **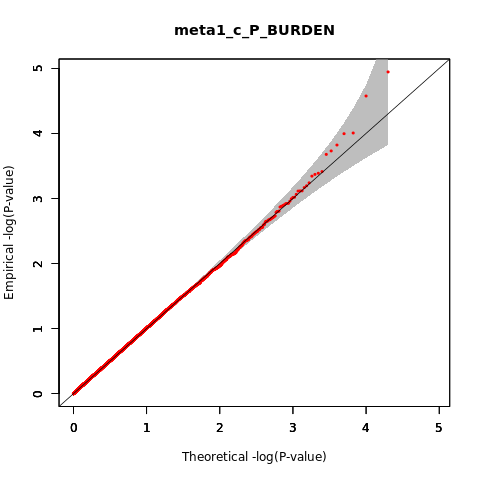** | **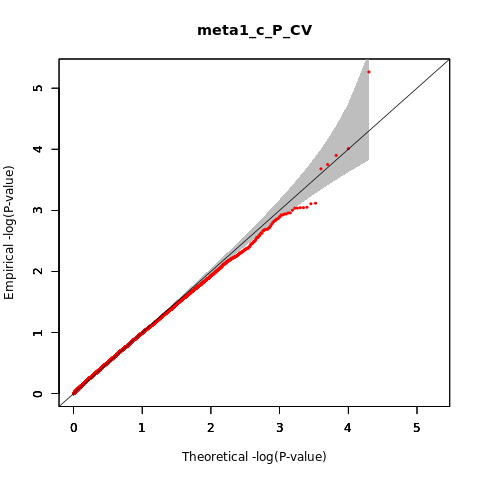** |
| 1. SKATO-type metaFARVAT^Het^ |  |
| 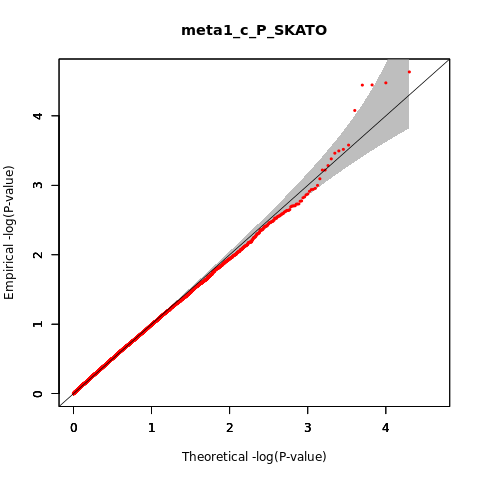 |  |

**Supplementary Figure 6: QQ plots for meta analyses of quantitative phenotype based on 6 studies** QQ plots were provided for results from the proposed methods under the null hypothesis. The empirical p-values were calculated under the null hypothesis with 20,000 replicates.

| 1. Burden-type metaFARVAT^Hom^ | 1. SKAT-type metaFARVAT^Hom^ |
| --- | --- |
| **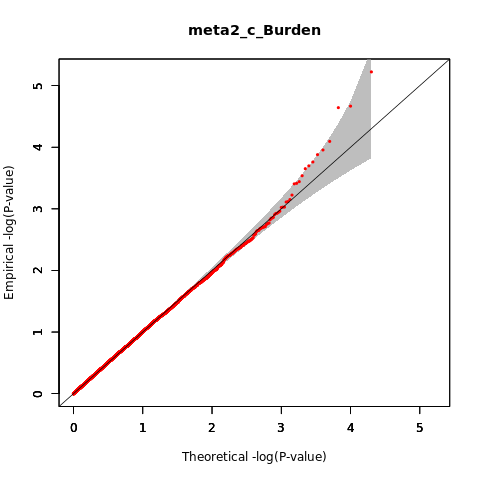** | **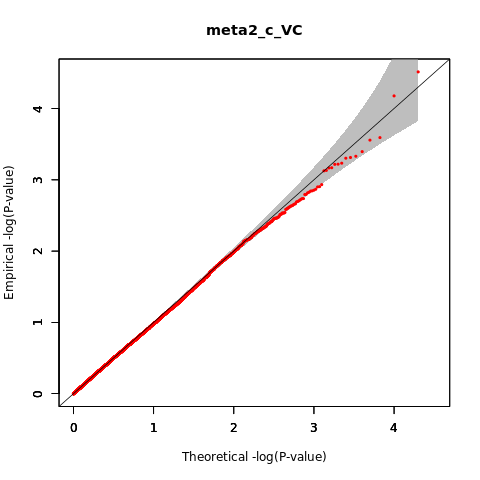** |
| 1. SKATO-type metaFARVAT^Hom^ | 1. VT-type metaFARVAT^Hom^ |
| 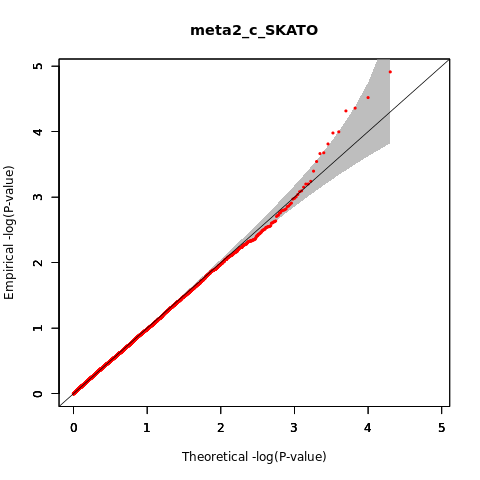 | 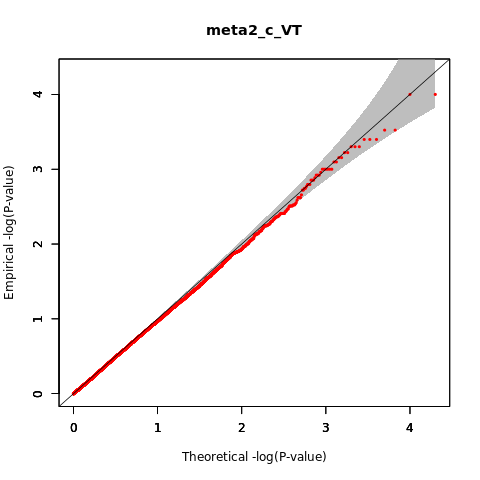 |
| 1. Burden-type metaFARVAT^Het^ | 1. SKAT-type metaFARVAT^Het^ |
| **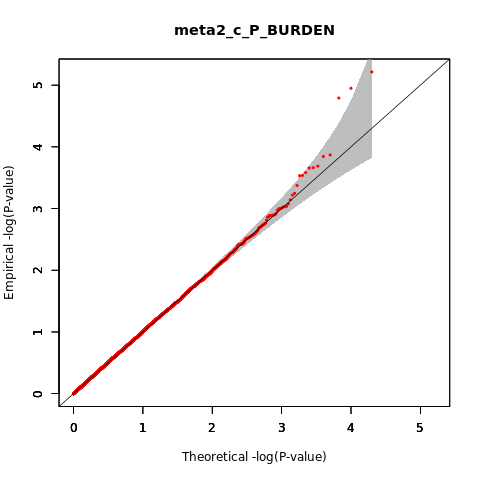** | **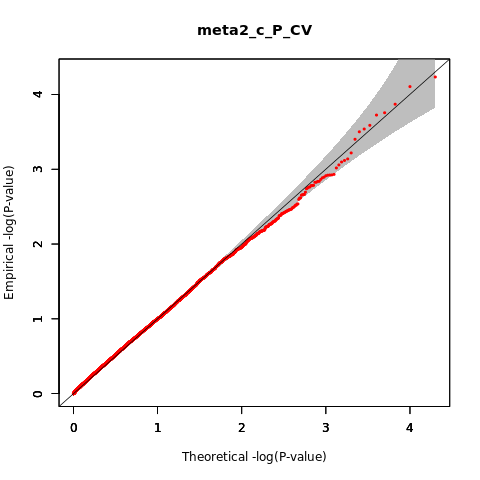** |
| 1. SKATO-type metaFARVAT^Het^ |  |
| 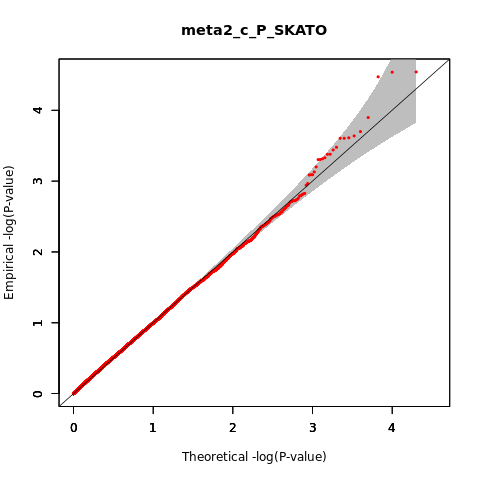 |  |

**Supplementary Figure 7: QQ plots for meta analyses of quantitative phenotype based on 9 studies** QQ plots were provided for results from the proposed methods under the null hypothesis. The empirical p-values were calculated under the null hypothesis with 20,000 replicates.

| 1. Burden-type metaFARVAT^Hom^ | 1. SKAT-type metaFARVAT^Hom^ |
| --- | --- |
| **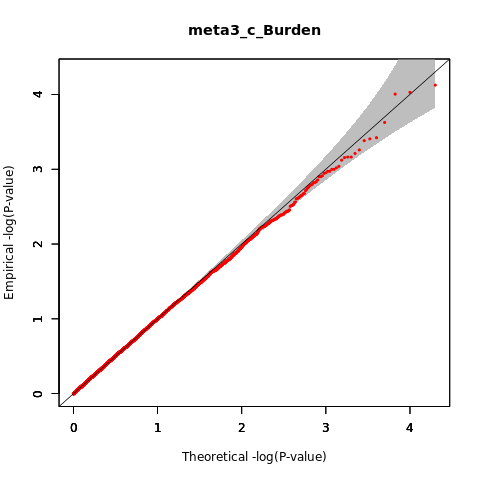** | **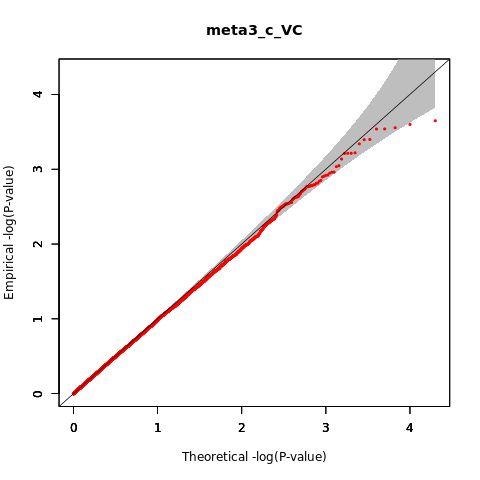** |
| 1. SKATO-type metaFARVAT^Hom^ | 1. VT-type metaFARVAT^Hom^ |
| 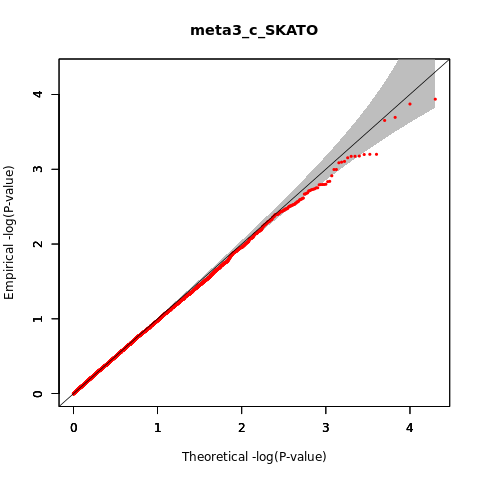 | 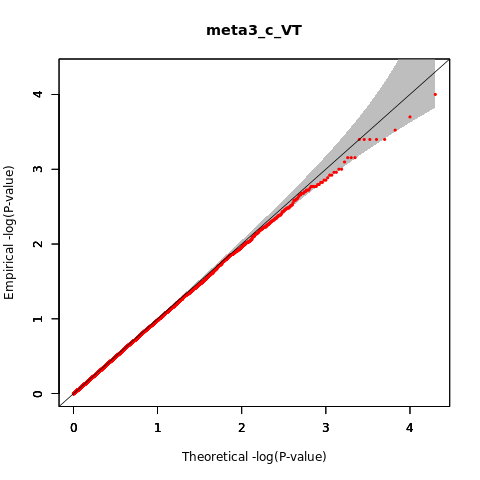 |
| 1. Burden-type metaFARVAT^Het^ | 1. SKAT-type metaFARVAT^Het^ |
| **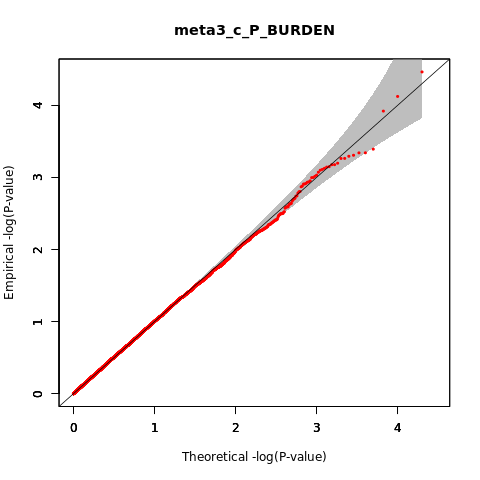** | **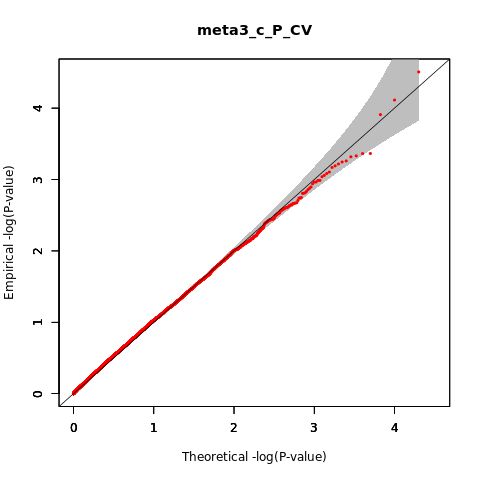** |
| 1. SKATO-type metaFARVAT^Het^ |  |
| 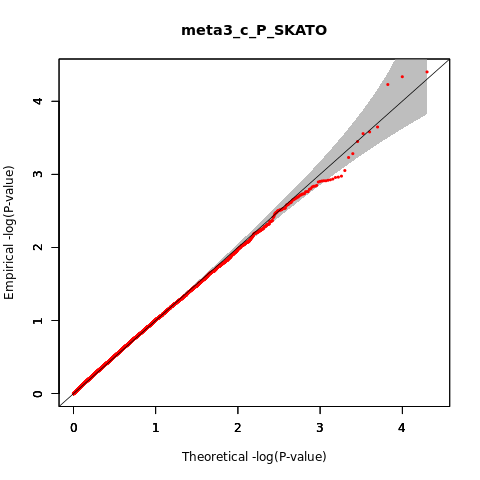 |  |

**Supplementary Figure 8: QQ plots and Manhattan plots are based on the results of the association analyses with EOCOPD and COPDGene datasets using FARVAT.** EOCOPD and COPDGene were separately analyzed using FARVAT and the results of SKAT-O type statistic were used for QQ plots and Manhattan plots**.** (a) and (b) are for EOCOPD dataset, and (c) and (d) are for COPDGene.

| 1. $FARVAT for EOCOPD: QQ plot$ | 1. $FARVAT for EOCOPD:Manhattan plot$ |
| --- | --- |
| **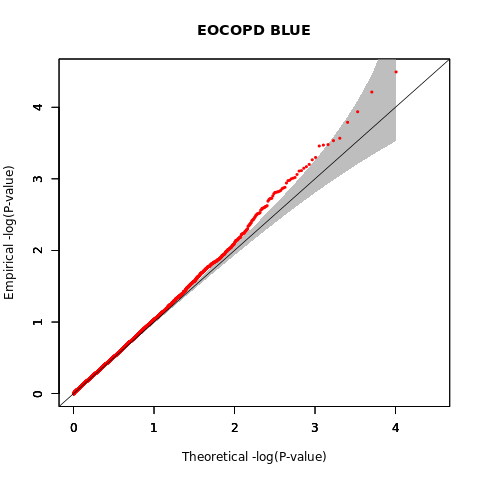** | **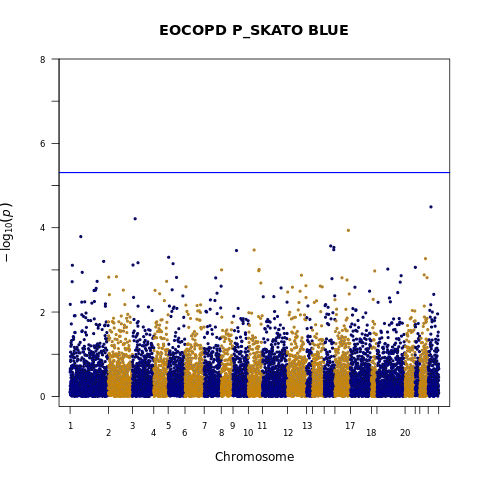** |
| 1. $FARVAT for COPDGene:QQ plot$ | 1. $FARVAT for COPDGene:Manhattan plot$ |
| 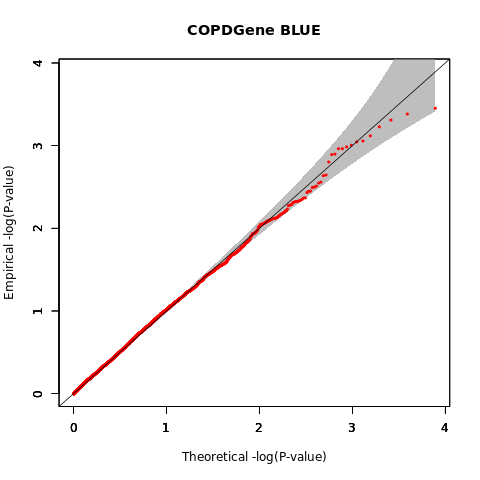 | 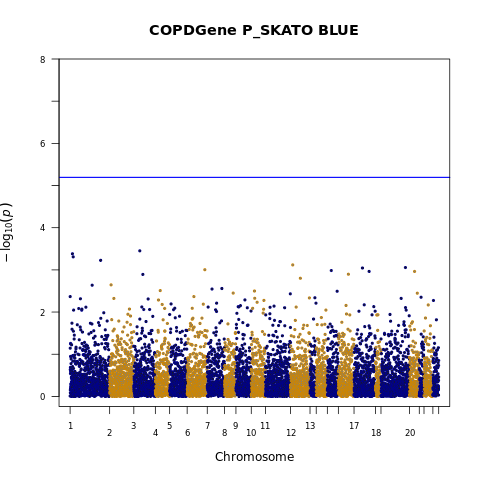 |

**Supplementary Figure 9: QQ plots of results from metaFARVAT with the EOCOPD and the COPDGene datasets.** metaFARVAT was applied to meta-analysis with the EOCOPD and the COPDGene datasets. (a), (b) and (c) were based on results from SKAT-O metaFARVAT^Hom^, SKAT-O metaFARVAT^Het^ and metaFARVAT^VT^ respectively.

| 1. metaFARVAT^Hom^ | 1. metaFARVAT^Het^ |
| --- | --- |
| **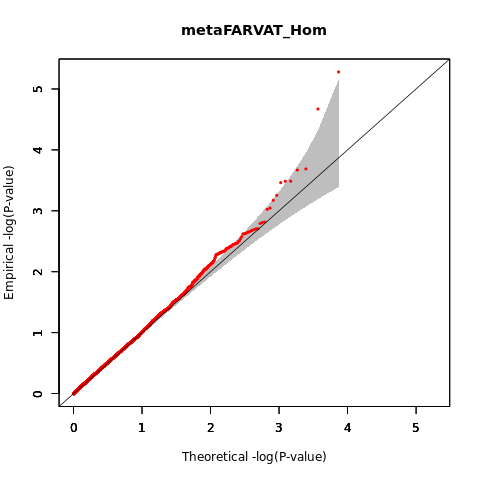** | **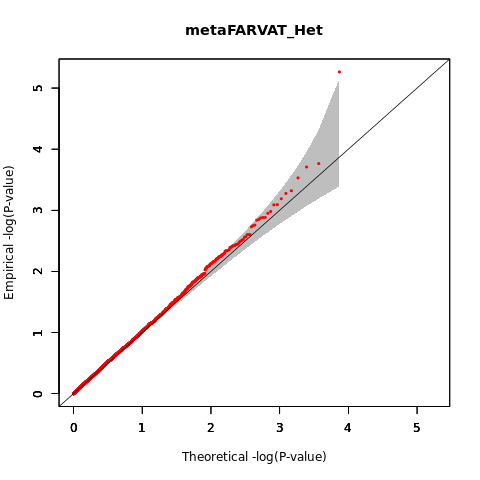** |
| 1. metaFARVAT^VT^ |  |
| 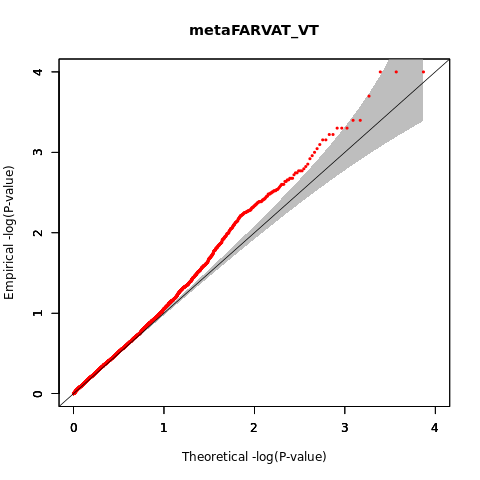 |  |

**Supplementary Figure 10: Manhattan plots of results from metaFARVAT with the EOCOPD and the COPDGene datasets.** metaFARVAT was applied to meta-analysis with the EOCOPD and the COPDGene datasets. (a), (b) and (c) were based on results from SKAT-O metaFARVAT^Hom^, SKAT-O metaFARVAT^Het^ and metaFARVAT^VT^ respectively.

| 1. metaFARVAT^Hom^ | 1. metaFARVAT^Het^ |
| --- | --- |
| **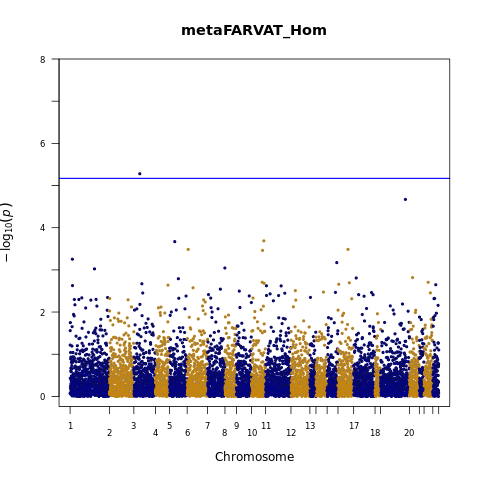** | **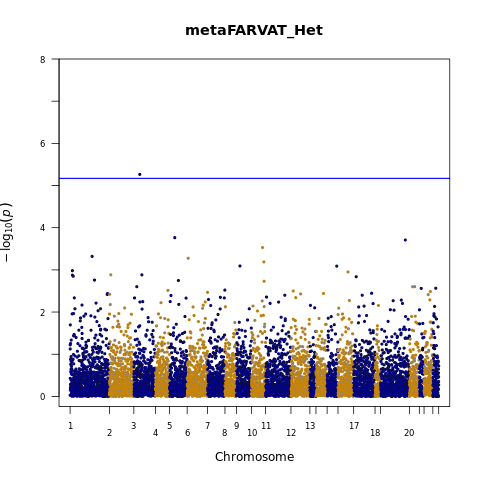** |
| 1. metaFARVAT^VT^ |  |
| 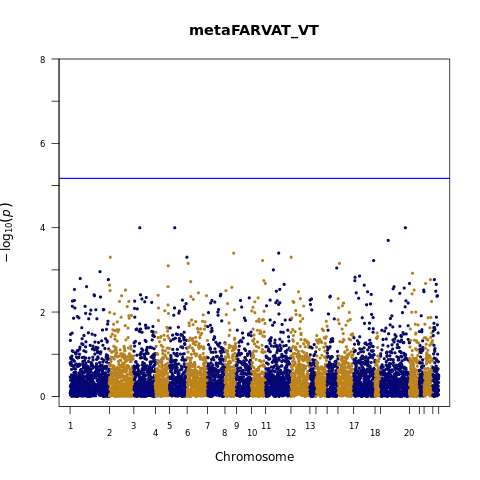 |  |

**Supplementary Table 1: Type-1 error estimates from simulation study for quantitative phenotypes.** The empirical type-1 error was estimated for proposed methods with 20,000 replicates at the 0.1, 0.01, 10^-3^ and 10^-4^ significance levels for quantitative phenotypes. We applied the proposed methods to meta analyses based on 3, 6 and 9 studies. We assumed that the number of rare variants is 60, and their minor allele frequencies <0.1.

|  |  |  | Quantitative | | | |
| --- | --- | --- | --- | --- | --- | --- |
|  | # studies | significance level | Burden | SKAT | SKAT-O | VT |
| metaFARVAT^Hom^ | 3 | 0.1 | 0.0989 | 0.0965 | 0.0993 | 0.1044 |
|  |  | 0.01 | 0.0094 | 0.0083 | 0.0094 | 0.0108 |
|  |  | 10^-3^ | 0.0012 | 0.0011 | 0.0009 | 0.0018 |
|  |  | 10^-4^ | 0.0001 | 0.0001 | 0.0002 | 0.0006 |
|  | 6 | 0.1 | 0.1010 | 0.0948 | 0.0959 | 0.0929 |
|  |  | 0.01 | 0.0092 | 0.0097 | 0.0094 | 0.0087 |
|  |  | 10^-3^ | 0.0010 | 0.0008 | 0.0010 | 0.0009 |
|  |  | 10^-4^ | 0.0002 | 0.0001 | 0.0002 | 0.0001 |
|  | 9 | 0.1 | 0.0999 | 0.0963 | 0.0948 | 0.0959 |
|  |  | 0.01 | 0.0094 | 0.0085 | 0.0090 | 0.0090 |
|  |  | 10^-3^ | 0.0008 | 0.0008 | 0.0007 | 0.0006 |
|  |  | 10^-4^ | 0.0002 | 0.0000 | 0.0000 | 0.0000 |
| metaFARVAT^Het^ | 3 | 0.1 | 0.1017 | 0.0984 | 0.0976 | -- |
|  |  | 0.01 | 0.0093 | 0.0084 | 0.0085 | -- |
|  |  | 10^-3^ | 0.0010 | 0.0007 | 0.0007 | -- |
|  |  | 10^-4^ | 0.0002 | 0.0001 | 0.0003 | -- |
|  | 6 | 0.1 | 0.1010 | 0.0996 | 0.0989 | -- |
|  |  | 0.01 | 0.0098 | 0.0091 | 0.0095 | -- |
|  |  | 10^-3^ | 0.0011 | 0.0008 | 0.0011 | -- |
|  |  | 10^-4^ | 0.0002 | 0.0001 | 0.0002 | -- |
|  | 9 | 0.1 | 0.1007 | 0.1072 | 0.1013 | -- |
|  |  | 0.01 | 0.0097 | 0.0101 | 0.0094 | -- |
|  |  | 10^-3^ | 0.0011 | 0.0008 | 0.0005 | -- |
|  |  | 10^-4^ | 0.0001 | 0.0001 | 0.0002 | -- |

**Supplementary Table 2. Empirical power estimates for meta-analyses of quantitative phenotype for homogeneous variants among studies.** Empirical power estimates of proposed methods for quantitative phenotypes were calculated with homogeneous effects at the 2.5×10^-6^ significant level. Empirical power esetimates of burden, SKAT and VT type of RAREMETAL and seqMeta were calculated with the same dataset and were compared with metaFARVAT method.

|  |  | 3 studies | | | | 6 studies | | | | 9 studies | | | |
| --- | --- | --- | --- | --- | --- | --- | --- | --- | --- | --- | --- | --- | --- |
| +/- | Method | SKAT | Burden | SKAT-O | VT | SKAT | Burden | SKAT-O | VT | SKAT | Burden | SKAT-O | VT |
| 60/0 | Fisher | 0.6300 | | | | 0.9870 | | | | 1.0000 | | | |
|  | minP | 0.1155 | | | | 0.1805 | | | | 0.2120 | | | |
|  | RAREMETAL | 0.0340 | 0.8330 | -- | 0.7535 | 0.4930 | 1.0000 | -- | 1.0000 | 0.9370 | 1.0000 | -- | 1.0000 |
|  | seqMeta | 0.0455 | 0.8655 | 0.8715 | -- | 0.5880 | 1.0000 | 1.0000 | -- | 0.9650 | 1.0000 | 1.0000 | -- |
|  | Hom | 0.0400 | 0.8650 | 0.8370 | 0.8700 | 0.5720 | 1.0000 | 1.0000 | 1.0000 | 0.9620 | 1.0000 | 1.0000 | 1.0000 |
|  | Het | 0.0030 | 0.8580 | 0.8605 | -- | 0.0465 | 1.0000 | 1.0000 | -- | 0.1850 | 1.0000 | 1.0000 | -- |
| 48/12 | Fisher | 0.0820 | | | | 0.4025 | | | | 0.7240 | | | |
|  | minP | 0.0090 | | | | 0.0185 | | | | 0.0185 | | | |
|  | RAREMETAL | 0.0295 | 0.1065 | -- | 0.0830 | 0.4750 | 0.5935 | -- | 0.5215 | 0.9285 | 0.9120 | -- | 0.8770 |
|  | seqMeta | 0.0440 | 0.1475 | 0.1640 | -- | 0.5740 | 0.6555 | 0.6855 | -- | 0.9545 | 0.9360 | 0.9525 | -- |
|  | Hom | 0.0400 | 0.1455 | 0.2345 | 0.1875 | 0.5580 | 0.6540 | 0.8705 | 0.6855 | 0.9505 | 0.9335 | 0.9955 | 0.9395 |
|  | Het | 0.0070 | 0.1465 | 0.2315 | -- | 0.0750 | 0.6440 | 0.7810 | -- | 0.2070 | 0.9295 | 0.9725 | -- |
| 30/30 | Fisher | 0.0035 | | | | 0.0225 | | | | 0.0690 | | | |
|  | minP | 0.0000 | | | | 0.0000 | | | | 0.0005 | | | |
|  | RAREMETAL | 0.0420 | 0.0000 | -- | 0.0010 | 0.4470 | 0.0000 | -- | 0.0025 | 0.9140 | 0.0000 | -- | 0.0110 |
|  | seqMeta | 0.0590 | 0.0000 | 0.0000 | -- | 0.5610 | 0.0000 | 0.0000 | -- | 0.9500 | 0.0000 | 0.0000 | -- |
|  | Hom | 0.0520 | 0.0000 | 0.0250 | 0.0025 | 0.5515 | 0.0000 | 0.4065 | 0.0070 | 0.9470 | 0.0000 | 0.8975 | 0.0250 |
|  | Het | 0.0125 | 0.0000 | 0.0050 | -- | 0.0845 | 0.0000 | 0.0530 | -- | 0.2430 | 0.0000 | 0.1535 | -- |
|  | Fisher | 0.1725 | | | | 0.6295 | | | | 0.9005 | | | |
|  | minP | 0.0195 | | | | 0.0270 | | | | 0.0335 | | | |
|  | RAREMETAL | 0.0540 | 0.2405 | -- | 0.1925 | 0.6015 | 0.8490 | -- | 0.7990 | 0.9635 | 0.9885 | -- | 0.9795 |
|  | seqMeta | 0.0605 | 0.2820 | 0.3095 | -- | 0.6510 | 0.8840 | 0.9015 | -- | 0.9720 | 0.9910 | 0.9945 | -- |
|  | Hom | 0.0535 | 0.2865 | 0.3900 | 0.3410 | 0.6385 | 0.8820 | 0.9490 | 0.8910 | 0.9705 | 0.9915 | 1.0000 | 0.9915 |
|  | Het | 0.0100 | 0.2830 | 0.3905 | -- | 0.0935 | 0.8735 | 0.9380 | -- | 0.2750 | 0.9900 | 0.9970 | -- |
| 24/6 | Fisher | 0.0175 | | | | 0.1295 | | | | 0.3445 | | | |
|  | minP | 0.0025 | | | | 0.0025 | | | | 0.0025 | | | |
|  | RAREMETAL | 0.0515 | 0.0100 | -- | 0.0070 | 0.5460 | 0.1145 | -- | 0.0995 | 0.9465 | 0.3650 | -- | 0.3290 |
|  | seqMeta | 0.0620 | 0.0150 | 0.0200 | -- | 0.6155 | 0.1490 | 0.1915 | -- | 0.9595 | 0.4335 | 0.5120 | -- |
|  | Hom | 0.0565 | 0.0145 | 0.0825 | 0.0315 | 0.6060 | 0.1490 | 0.6478 | 0.2045 | 0.9565 | 0.4305 | 0.9720 | 0.4805 |
|  | Het | 0.0130 | 0.0125 | 0.0615 | -- | 0.0795 | 0.1410 | 0.3690 | -- | 0.2335 | 0.4120 | 0.7245 | -- |
| 15/15 | Fisher | 0.0010 | | | | 0.0185 | | | | 0.0655 | | | |
|  | minP | 0.0000 | | | | 0.0005 | | | | 0.0000 | | | |
|  | RAREMETAL | 0.0360 | 0.0000 | -- | 0.0000 | 0.4420 | 0.0000 | -- | 0.0010 | 0.9065 | 0.0000 | -- | 0.0070 |
|  | seqMeta | 0.0485 | 0.0000 | 0.0000 | -- | 0.5525 | 0.0000 | 0.0000 | -- | 0.9470 | 0.0000 | 0.0000 | -- |
|  | Hom | 0.0470 | 0.0000 | 0.0190 | 0.0000 | 0.5420 | 0.0000 | 0.3875 | 0.0070 | 0.9470 | 0.0000 | 0.8825 | 0.0270 |
|  | Het | 0.0070 | 0.0000 | 0.0035 | -- | 0.0810 | 0.0000 | 0.0515 | -- | 0.2420 | 0.0000 | 0.1570 | -- |

**Supplementary Table 3. Empirical power estimates for meta-analyses of quantitative phenotype for heterogeneous variants among studies.** Empirical power estimates of burden, SKAT, SKAT-O and VT type of metaFARVAT^Hom^ and metaFARVAT^Het^ were calculated for quantitative phenotypes with heterogeneous effects at the 2.5×10^-6^ significant level. Empirical power estimates of burden, SKAT and VT type of RAREMETAL and seqMeta were calculated with the same dataset and were compared with metaFARVAT method.

|  |  | 3 studies | | | | 6 studies | | | | 9 studies | | | |
| --- | --- | --- | --- | --- | --- | --- | --- | --- | --- | --- | --- | --- | --- |
| +/- | Method | SKAT | Burden | SKAT-O | VT | SKAT | Burden | SKAT-O | VT | SKAT | Burden | SKAT-O | VT |
| 48/12 | Fisher | 0.0720 | | | | 0.3765 | | | | 0.7120 | | | |
|  | minP | 0.0080 | | | | 0.0085 | | | | 0.0130 | | | |
|  | RAREMETAL | 0.0190 | 0.2675 | -- | 0.2015 | 0.1405 | 0.8720 | -- | 0.8050 | 0.4410 | 0.9910 | -- | 0.9825 |
|  | seqMeta | 0.0095 | 0.1270 | 0.1410 | -- | 0.0560 | 0.6205 | 0.6400 | -- | 0.2345 | 0.9345 | 0.9390 | -- |
|  | Hom | 0.0085 | 0.1270 | 0.1515 | 0.1740 | 0.0580 | 0.6190 | 0.6525 | 0.6675 | 0.2290 | 0.9335 | 0.9410 | 0.9465 |
|  | Het | 0.0045 | 0.1205 | 0.2040 | -- | 0.0615 | 0.6065 | 0.7650 | -- | 0.2075 | 0.9255 | 0.9700 | -- |
| 30/30 | Fisher | 0.0050 | | | | 0.0190 | | | | 0.0565 | | | |
|  | minP | 0.0000 | | | | 0.0000 | | | | 0.0005 | | | |
|  | RAREMETAL | 0.0015 | 0.0000 | -- | 0.0000 | 0.0030 | 0.0000 | -- | 0.0005 | 0.0015 | 0.0000 | -- | 0.0000 |
|  | seqMeta | 0.0020 | 0.0005 | 0.0005 | -- | 0.0015 | 0.0000 | 0.0000 | -- | 0.0020 | 0.0000 | 0.0000 | -- |
|  | Hom | 0.0020 | 0.0005 | 0.0005 | 0.0015 | 0.0015 | 0.0000 | 0.0005 | 0.0005 | 0.0025 | 0.0000 | 0.0005 | 0.0000 |
|  | Het | 0.0130 | 0.0005 | 0.0070 | -- | 0.0750 | 0.0000 | 0.0440 | -- | 0.2265 | 0.0000 | 0.1370 | -- |
| 30/0 | Fisher | 0.1825 | | | | 0.6635 | | | | 0.9060 | | | |
|  | minP | 0.0230 | | | | 0.0295 | | | | 0.0350 | | | |
|  | RAREMETAL | 0.0190 | 0.2675 | -- | 0.2015 | 0.1405 | 0.8720 | -- | 0.8050 | 0.4410 | 0.9910 | -- | 0.9825 |
|  | seqMeta | 0.0135 | 0.3025 | 0.3175 | -- | 0.1680 | 0.8875 | 0.8955 | -- | 0.4975 | 0.9945 | 0.9965 | -- |
|  | Hom | 0.0125 | 0.3005 | 0.3245 | 0.3540 | 0.1575 | 0.8855 | 0.8875 | 0.8995 | 0.4800 | 0.9950 | 0.9925 | 0.9945 |
|  | Het | 0.0115 | 0.2865 | 0.4045 | -- | 0.0960 | 0.8770 | 0.9295 | -- | 0.2530 | 0.9915 | 0.9985 | -- |
| 24/6 | Fisher | 0.0215 | | | | 0.1395 | | | | 0.3550 | | | |
|  | minP | 0.0025 | | | | 0.0035 | | | | 0.0050 | | | |
|  | RAREMETAL | 0.0035 | 0.0180 | -- | 0.0110 | 0.0225 | 0.1595 | -- | 0.1080 | 0.0575 | 0.4230 | -- | 0.3390 |
|  | seqMeta | 0.0035 | 0.0225 | 0.0245 | -- | 0.0190 | 0.1745 | 0.1900 | -- | 0.0600 | 0.4435 | 0.4630 | -- |
|  | Hom | 0.0030 | 0.0220 | 0.0360 | 0.0395 | 0.0175 | 0.1710 | 0.2270 | 0.2240 | 0.0600 | 0.4430 | 0.5000 | 0.5090 |
|  | Het | 0.0075 | 0.0200 | 0.0605 | -- | 0.0750 | 0.1560 | 0.3715 | -- | 0.2460 | 0.4110 | 0.7275 | -- |
| 15/15 | Fisher | 0.0020 | | | | 0.0200 | | | | 0.0615 | | | |
|  | minP | 0.0005 | | | | 0.0000 | | | | 0.0000 | | | |
|  | RAREMETAL | 0.0025 | 0.0000 | -- | 0.0000 | 0.0015 | 0.0000 | -- | 0.0000 | 0.0035 | 0.0000 | -- | 0.0000 |
|  | seqMeta | 0.0040 | 0.0000 | 0.0000 | -- | 0.0005 | 0.0000 | 0.0000 | -- | 0.0035 | 0.0000 | 0.0000 | -- |
|  | Hom | 0.0035 | 0.0000 | 0.0000 | 0.0010 | 0.0005 | 0.0000 | 0.0005 | 0.0000 | 0.0040 | 0.0000 | 0.0005 | 0.0015 |
|  | Het | 0.0130 | 0.0000 | 0.0090 | -- | 0.0700 | 0.0000 | 0.0485 | -- | 0.2380 | 0.0000 | 0.1485 | -- |

**Supplementary Table 4. The description of COPD datasets, EOCOPD WES and COPDGene.** This description includes the range of age, and the number of individuals, families, females/males, cases/controls/missing, variants, rare variants (MAF <5% in dbSNP) and genes.

|  | EOCOPD WES | COPDGene |
| --- | --- | --- |
| age | [21, 87] | [46, 81] |
| Sample size | 303 | 394 |
| Families | 49 | -- |
| F/M | 209/138 | 211/200 |
| Cases/controls | 155/148 | 204/195 |
| Variants | 124,288 | 108,443 |
| Rare variants | 88,373 | 24,846 |
| Genes | 13,935 | 10,550 |
